# Meneco, a Topology-Based Gap-Filling Tool Applicable to Degraded Genome-Wide Metabolic Networks

**DOI:** 10.1371/journal.pcbi.1005276

**Published:** 2017-01-27

**Authors:** Sylvain Prigent, Clémence Frioux, Simon M. Dittami, Sven Thiele, Abdelhalim Larhlimi, Guillaume Collet, Fabien Gutknecht, Jeanne Got, Damien Eveillard, Jérémie Bourdon, Frédéric Plewniak, Thierry Tonon, Anne Siegel

**Affiliations:** 1 Institute for Research in IT and Random Systems - IRISA, Université de Rennes 1, Rennes, France; 2 Department of Biology and Biological Engineering, Chalmers University of Technology, Göteborg, Sweden; 3 Irisa, CNRS, Rennes, France; 4 Dyliss, Inria, Rennes, France; 5 Sorbonne Universités, UPMC Univ Paris 06, CNRS, UMR 8227, Integrative Biology of Marine Models, Station Biologique de Roscoff, Roscoff, France; 6 Computer Science Laboratory of Nantes Atlantique - LINA UMR6241, Université de Nantes, Nantes, France; 7 Molecular Genetics, Genomics and Microbiology - GMGM, Université de Strasbourg, Strasbourg, France; 8 GMGM, CNRS, Strasbourg, France; Christian Albrechts Universitat zu Kiel, GERMANY

## Abstract

Increasing amounts of sequence data are becoming available for a wide range of non-model organisms. Investigating and modelling the metabolic behaviour of those organisms is highly relevant to understand their biology and ecology. As sequences are often incomplete and poorly annotated, draft networks of their metabolism largely suffer from incompleteness. Appropriate gap-filling methods to identify and add missing reactions are therefore required to address this issue. However, current tools rely on phenotypic or taxonomic information, or are very sensitive to the stoichiometric balance of metabolic reactions, especially concerning the co-factors. This type of information is often not available or at least prone to errors for newly-explored organisms. Here we introduce Meneco, a tool dedicated to the topological gap-filling of genome-scale draft metabolic networks. Meneco reformulates gap-filling as a qualitative combinatorial optimization problem, omitting constraints raised by the stoichiometry of a metabolic network considered in other methods, and solves this problem using Answer Set Programming. Run on several artificial test sets gathering 10,800 degraded *Escherichia coli* networks Meneco was able to efficiently identify essential reactions missing in networks at high degradation rates, outperforming the stoichiometry-based tools in scalability. To demonstrate the utility of Meneco we applied it to two case studies. Its application to recent metabolic networks reconstructed for the brown algal model *Ectocarpus siliculosus* and an associated bacterium *Candidatus* Phaeomarinobacter ectocarpi revealed several candidate metabolic pathways for algal-bacterial interactions. Then Meneco was used to reconstruct, from transcriptomic and metabolomic data, the first metabolic network for the microalga *Euglena mutabilis*. These two case studies show that Meneco is a versatile tool to complete draft genome-scale metabolic networks produced from heterogeneous data, and to suggest relevant reactions that explain the metabolic capacity of a biological system.

## Author Summary

In the era of fast and massive genome sequencing, one challenge is to transform sequence information into biological knowledge. Reconstructing metabolic networks that include all biochemical reactions of a cell is a way to infer reactions from genomic data. Unfortunately, those data are usually incomplete, poorly annotated, and missing reactions create gaps in the metabolic networks. Here we introduce Meneco, a tool dedicated to the parsimonious gap-filling of metabolic networks. Unlike other tools, Meneco allows using sparse data (missing stoichiometries) and draft metabolic networks to suggest reactions to fill gaps in the networks. Subsequently, we apply it to two biological case studies and show that the flexibility of Meneco enables it to be adapted to a variety of research questions and types of available data. We show that Meneco performs better than reference tools with respect to large-scale heterogeneous reference database and with respect to the recovery of important reactions in highly degraded networks. Specifically, it allowed the analysis of two interacting metabolic networks and the reconstruction of the first metabolic network of *Euglena mutabilis*.

## Introduction

### Gap-filling a metabolic network: A very sensitive approach

Metabolic knowledge is crucial to understand physiology and biotic interactions. Supported by an unprecedented rise of sequencing technologies, the last decade saw the increasing understanding of metabolic capacities using genomic knowledge. In particular, in 2010, Thiele and Palsson [1] described a general protocol enabling the reconstruction of high-quality metabolic networks, and several approaches have since been proposed to automate this process [2–4]. These methods rely primarily on two distinct steps. First, they provide automatic reconstructions of networks, called draft metabolic networks [5, 6], and in a second step fill the gaps of the draft networks. To this end, reference databases of metabolic reactions are used to check whether adding reactions to networks allows compounds of interest to be produced from given growth media. Identifying these missing reactions constitutes the so-called *gap-filling* problem (*n.b.* considering the diversity of compound producibility and network consistency definitions, we here refer to a family of gap-filling problems rather than a single one) [2].

Several approaches exist to solve gap-filling problems and to select, within databases, those reactions that must be added to the draft network to restore its consistency and a metabolic behavior. Reactions may be chosen to optimize a graph-based criterion [7], or to optimize a linear score modelling the quantitative metabolic production of the system, as in the GapFill tool [8], and its derivative fastGapFill [9]. Some approaches also integrate complementary knowledge such as taxonomic information [10] or compartment modularity [11]. More generally, the selection of reactions may be performed by optimizing a linear score modelling the consistency of a network with phenotypic knowledge, *i.e.* experimental flux data [12] or growth/no growth results [13]. Lastly, some tools combine several of the previously mentioned approaches. An example of these is MIRAGE, which selects reactions in a database in order to maintain biomass producibility with respect to a score based on co-expression and taxonomic distance between the target species and the species for which enzymes were evidenced [14]. The approach presented in [15] is similar although based on a different definition of producibility. In Table in S1 Table, we report the main characteristics of such methods in terms of required input data, the technological platform to be run, and examples of applications. Together these examples illustrate that methods to reconstruct metabolic networks have been very fruitful. However, as noted in [16], most published genome-scale metabolic networks (GEMs) concern either prokaryotic or eukaryotic organisms for which genomic and physiological knowledge results from years of intensive studies. Indeed, GEM reconstruction is very sensitive to both genome annotation and the availability of complementary knowledge. It cannot, for instance, take into account genes of unknown function, which are common in incomplete or roughly annotated genomes.

### Issues raised by the application of gap-filling methods to degraded metabolic networks

Nowadays, next generation sequencing (NGS) technologies are commonly employed to study strains and species distantly related to common model organisms. Draft metabolic networks based on these technologies are frequently quite degraded compared to those for standard model organisms. For instance, when comparing the number of reactions in the BioCyc repository [17] version 19.5, we noticed that the 7,296 automatically reconstructed bacterial networks (Tier 3) contained on average 8% fewer reactions than the 27 curated bacterial metabolic networks contained in the manually curated repositories (Tier 1 & Tier 2). For the sake of illustration, let us introduce two examples of organisms with a complex evolutionary history and recently studied using NGS technologies.

*Euglena mutabilis* is a photosynthetic protist and important primary producer in acidic aquatic environments. Despite the crucial role of *E. mutabilis* in these ecosystems and the fact that it has often been considered as an indicator species for acid mine drainages (AMDs), this organism has only been poorly described so far, in contrast to another species of the same genus, *Euglena gracilis*. The available data for *E. mutabilis* consists in assembled transcript sequences obtained from *de novo* transcriptomics and metabolomics experiments previously published in [18] and in [19], respectively. This sparse dataset prevented us from using most of the tools described above to construct a metabolic network: the absence of a sequenced genome for the *Euglena* genus and the fact that this genus is not closely related to any common model organism rendered taxonomy-based methods of network reconstruction unusable [10, 11]. *E. mutabilis* is difficult to cultivate in controlled conditions and to obtain as clonal cultures, preventing the use of phenotype-based tools [12, 13]. The family of tools [8, 9, 14] that could be used here are of functional nature.

Another application for gap-filling methods has emerged as a tool for studying the coexistence of organisms living in communities. As an example, *Candidatus* Phaeomarinobacter ectocarpi is a symbiotic bacterium associated with *Ectocarpus siliculosus*. Its genome and a draft of its metabolism could be produced [20]. Its host *E. siliculosus* has been studied for a longer time, and a functional metabolic network was reconstructed to explain the production of characteristic compounds of its metabolic profile [21]. Additional transcriptomic datasets were used in this study to identify 1,125 internal or external compounds produced by at least one reaction of *E. siliculosus* for which the corresponding enzyme was transcribed. Only 317 compounds could be produced according to the *E. siliculosus* draft network. In the framework of systems ecology [22], a natural question is whether the symbiotic bacterial network can resolve some of this non-producibility. This issue can be rephrased as follows: how can the *Candidatus* Phaeomarinobacter ectocarpi metabolic network be used to fill gaps in the *E. siliculosus* draft GEM? As above, this issue is of functional nature, and can be addressed only with functional gap-filling methods.

Let us point out, however, that applying functional GEM gap-filling techniques [8, 9, 14] to organisms distantly related to common model organisms raises several problems. A first problem is related to the determination of the biomass reaction of the system. This reaction is often copied from well-established model organisms and therefore cannot capture all of the characteristics of the studied organism, especially when dealing with extremophiles. Shortcomings in the determination of an adequate biomass reaction lead to a second problem, that is, the determination of the boundary compounds, dead-end metabolites, and cofactors in the system. These may be hard to characterize from experiments or literature, despite their strong potential impact on the capacity of the system to produce biomass according to stoichiometry-based formalisms [8]. In particular, the score-based methods mentioned above depend on the stoichiometric balance of metabolic reactions, a criterion which may be prone to errors, especially with respect to cofactors and when using large-scale databases of metabolic reactions [2].

### Meneco: A gap-filling method based on topological criteria enabling the identification of essential reactions

As a natural consequence, we advocate the need of GEM gap-filling techniques suitable for newly developed model organisms, in particular those with a complex evolutionary history and/or living in extreme environments for which phenotypic data are lacking. This study reformulates the gap-filling problem as a qualitative combinatorial (optimization) one. We introduce the tool Meneco (**Me**tabolic **Ne**twork **Co**mpletion) that solves this problem, using Answer Set Programming (ASP), a declarative programming paradigm including SAT-based solving technologies. Meneco considers reactions as achievable only if all their reactants are available, either as nutrients or provided by other metabolic reactions. Starting from given nutrients (*e.g.* growth medium), referred to as seeds, this tool computes their scope defined as all the metabolites that can be synthesized from them using a graph-based approach. For metabolic network gap-filling, a database of metabolic reactions is queried to look for minimal sets of reactions that can restore the observed bio-synthetic behaviour (*i.e* producibility of target metabolites).

The Meneco tool was included in a pipeline implemented to construct EctoGEM, a metabolic network for the brown algal model *E. siliculosus*. The analysis of EctoGEM highlighted several interesting biochemical reactions, shedding light on the organization and evolution of some primary metabolic pathways of photosynthetic organisms [21]. In the present work, the case for Meneco as an important tool for hypothesis generation is further supported by new observations related to a benchmark of networks on a model organism and two case studies. First, this study simulates different degrees of manual curation using the model *Escherichia coli* [1]. For this purpose, 3,600 metabolic networks were generated from randomly degraded *E. coli* metabolic networks. In this benchmark, when the reference database used for completion was the real-case study Metacyc, Meneco outperformed the GapFill, the fastGapFill and the MIRAGE algorithms in terms of performance or accuracy. On a larger benchmark of 10,800 metabolic networks, our analysis suggests that Meneco is functionally relevant by identifying all essential reactions for more than 95% of the degraded networks. We advocate that the identification of such essential reactions is a key step towards the understanding of the metabolic capabilities of the species of interest, because they are related to key enzymes which, when removed, most likely prevent the viability of the species. Our results show that, when focusing on networks with a 10% degradation rate, the Meneco tool is able to restore the functionality of the network in 82% of cases. This suggests that Meneco is an important tool to study metabolic networks produced for organisms distantly related to common model organisms.

In our first case study we use Meneco to assess the capability of the EctoGEM metabolic network to exchange metabolites with *Candidatus* Phaeomarinobacter ectocarpi, the aforementioned symbiotic bacterium associated with *E. siliculosus*. Combining the metabolic capacities of the draft GEM of *E. siliculosus* with those of the bacterial network enabled the *in-silico* production of 83 previously non producible algal targets. All of them were studied in detail allowing us to put forward hypotheses on possible exchanges between both organisms.

Our second case study presents the first metabolic network for *E. mutabilis*, based on transcript sequences assembled from previously published transcriptomic and metabolomic data [18] [19]. In order to complete this draft with Meneco, we selected a set of targets from the list of metabolites that *E. mutabilis* can accumulate or secrete in minimum mineral medium [19]. Except for cobalamine, a cofactor that is not produced by this organism but is required for methionine synthesis, *E. mutabilis* can grow on a strictly mineral medium and is able to synthesize all the basic components of its biomass from mineral compounds only. Gaps in the draft network were filled iteratively with Meneco, using for each iteration a different subset of the 72 targets to solve the problem of cycles and circular dependencies. We thus obtained a network which was functional in Flux Balance analysis (FBA) for the photosynthetic production of biomass and excreted metabolites.

## Results

### Handling a real-scale reference completion database with gap-filling algorithms

#### The gap-filling problem

Starting point for any metabolic gap-filling problem [23] is a draft GEM consisting of reactions and metabolites. A set of target metabolites (targets) that were experimentally shown to be produced by the organism of interest, possibly described as a linear combination of fluxes (e.g., a biomass function), is used. A set of nutrients (seeds) describes the growth medium. Finally, a pool of metabolic reactions (reference database) is assumed to be available to fill the network. We assume that the draft GEM contains at least one blocked reaction with respect to the production of the targeted compounds from the seeds. In other word, there exists at least one targeted compound which does not admit a non-zero steady state flux. A gap-filling method reports at least one reaction from the reference database that needs to be added such that at least one formerly blocked reaction can carry flux.

Obviously, the solution to a gap-filling problem may not be unique. The sizes of both of the search space and the solution space for the gap-filling problem grow exponentially with the size of the reference database from which the reactions are taken. Any gap-filling method thus needs to find a suitable trade-off between the number of solutions reported by the method, that should not be too large to enable a manual curation, and their biological significance, in order to capture the main properties of the GEM being reconstructed. Gap-filling methods may report one, a few, or many sets of reactions to complete the draft GEM. When several solutions are produced by a gap-filling algorithm, there are no *a priori* criteria to select one rather than the other. Then, a cautious strategy is to compute the set of all reactions contained in at least one solution reported by the algorithm and to manually curate them.

#### Stoichiometry-based heuristics to approximate gap-filling solutions

**Fig 1:**
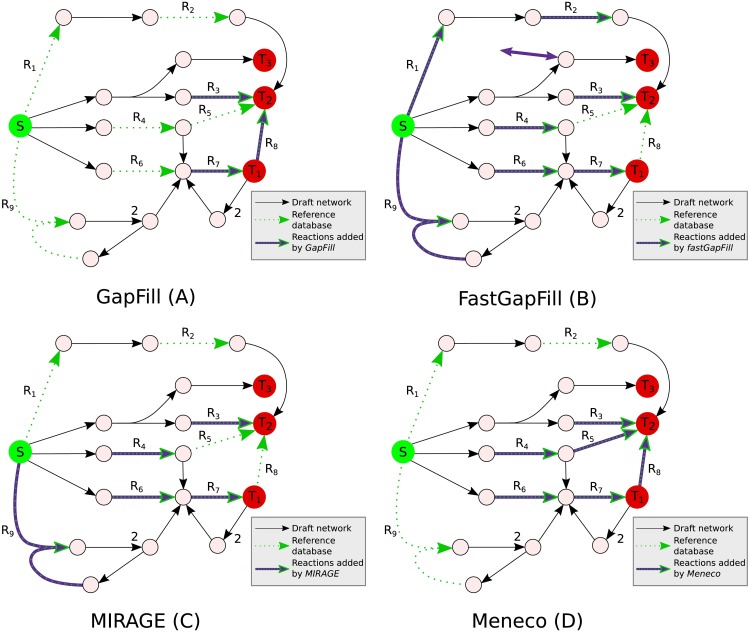
Gap-filling of metabolic networks with different heuristics. The reactions of the initial network are depicted as black arrows. Seeds (*e.g.* growth medium) and targets are *S* and *T* circles, respectively. The labels on the arrows depict the stoichiometry of the reactions. Green dotted arrows represent reactions that can be added to the network (reference database). The purple arrows represent reactions proposed by different gap-filling tools. GapFill (A) reported two reactions as a minimal completion and two different combinations to produce biomass from *T*_1_, *T*_2_ and *T*_3_, {*R*_3_, *R*_7_} and {*R*_7_, *R*_8_}. fastGapFill (B) reports one unique set of seven reactions to unblock all reactions of the example: {*R*_1_, *R*_2_, *R*_3_, *R*_4_, *R*_6_, *R*_7_, *R*_9_}. It also add an import/export reaction for the reactant of the reaction producing *T*_3_. In additions, 100 runs of MIRAGE (C) without scoring of reactions reported the following set of five reactions: {*R*_3_, *R*_4_, *R*_6_, *R*_7_, *R*_9_}. Finally, Meneco (D) reported that three reactions are needed to restore the topological producibility of the three targets, with five different combinations. Therefore, the output of Meneco is the set of six reactions {*R*_3_, *R*_4_, *R*_5_, *R*_6_, *R*_7_, *R*_8_}.

Three main approaches can be distinguished according to the number of solutions they report and the characteristics of these solutions (see their main features in S1 Appendix). The associated tools were run on a toy example depicted in Fig 1, to produce the biomass constituents *T*_1_, *T*_2_, *T*_3_.

GapFill uses a parsimonious bottom-up strategies [8, 10], which enriches the draft metabolic network until the targeted properties are satisfied. More precisely, GapFill detects a minimal number of reactions from the reference database to enable the synthesis of the targeted compounds according to stoichiometry-based producibility constraints. Importantly, GapFill allows the accumulation of internal compounds in the model. Applied to our toy example, GapFillreports a minimum of two reactions to enable the biomass synthesis. There exist two alternative sets of two reactions that do so: {*R*_3_, *R*_7_} and {*R*_7_, *R*_8_}. The union of reactions does not contain the reactions *R*_1_ and *R*_2_ since the parsimonious assumption omits alternative long pathways. The reaction *R*_7_ is used by GapFill to fill the cycle it is part of and to produce *T*_1_. Then GapFill uses either the reaction *R*_8_ or the reaction *R*_3_ to produce *T*_2_ since it considers *T*_3_ as already producible despite a possible accumulation of the reactant of *R*_3_. One limitation of this approach is that it reports a bounded (parameterized) number of solutions to the problem, although there is no a priori estimation of the number of iterations needed to solve the problem.

fastGapFill [9] is an extension of the GapFill approach combined with the Fastcore algorithm, which eliminates the focus on the biomass production by identifying a single set of reactions which unblocks all reactions within the draft metabolic network. The price to pay is to allow import and export fluxes, especially to resolve issues related to the activation of isolated reactions in the GEM. When applied to our toy example, fastGapFill reported seven reactions from the reference database and one import/export reaction of the reactant of the reaction producing *T*_3_ that was not present in the database to be added to the model. The reactions *R*_1_ and *R*_2_ are introduced to produce *T*_2_ in the absence of *R*_3_. Since no accumulation of the reactant of *R*_3_ is possible, fastGapFill adds an import/export reaction to enable the production of *T*_3_ without using the previous reactions of this pathway. The reactions *R*_4_, *R*_6_, and *R*_9_ are equivalent to produce *T*_1_ and were all chosen in order to unblock all fluxes going threw all reactions present in the draft model (including the vertical reaction going from the product of *R*_4_ to the reactant of *R*_7_). Finally, *R*_7_ is added to enable the production of *T*_1_.

In contrast to parsimonious approaches, top-down approaches start from all available information and remove reactions without added-value to the solution of the problem [15, 23, 24]. Among them, MIRAGE [14] aims at relaxing the minimality condition over the number of added reactions by identifying all subset minimal sets of reactions which enable the functionality of the biomass reaction. A subset-minimal reaction set might include of a higher number of reactions but it is minimal in the sense that it loses its capability to restore biomass functionality as soon as any one reaction is removed from the set. As the number of such sets of reactions increases dramatically with the size of the reference database, MIRAGE samples the space of solutions by randomly iterating the search algorithm. In our toy example, the algorithm was iterated 100 times without applying *a priori* scores to reactions of the database. Four different sets of reactions were obtained {*R*_3_, *R*_7_}, {*R*_3_, *R*_6_, *R*_7_}, {*R*_3_, *R*_4_, *R*_7_} and {*R*_3_, *R*_7_, *R*_9_}. The reaction *R*_3_ is mandatory to produce *T*_3_ and it also produces *T*_2_. Therefore, no other reaction producing *T*_2_ is necessary, so that *R*_1_, *R*_2_, *R*_5_ and *R*_8_ were never used. Given the cycle, the reaction *R*_7_ would be the minimal completion to produce *T*_1_ from a stoichiometric point of view, but the subset-minimality criterion also enables the MIRAGE algorithm to find the reactions *R*_4_, *R*_6_ and *R*_7_.

Together, we noticed that the criteria used to complete the network highly impact on the reconstruction procedures which all report different solutions. In particular, the reaction *R*_5_ is identified by none of the stoichiometry-based methods.

#### Meneco: Using a topological over-approximation to enable an exhaustive enumeration of parsimonious solutions

Stoichiometry-based tools fail to address the fact that species-specificity of cofactors in the reactions is often not precisely described in databases neither is stoichiometry of reactions. This may lead to predictions errors for degraded metabolic networks while producing biomass. Topology-based tools on the other hand do not take into account constraints yielded by the stoichiometry that may lead to non-producibility of biomass in some cases.

**Fig 2:**
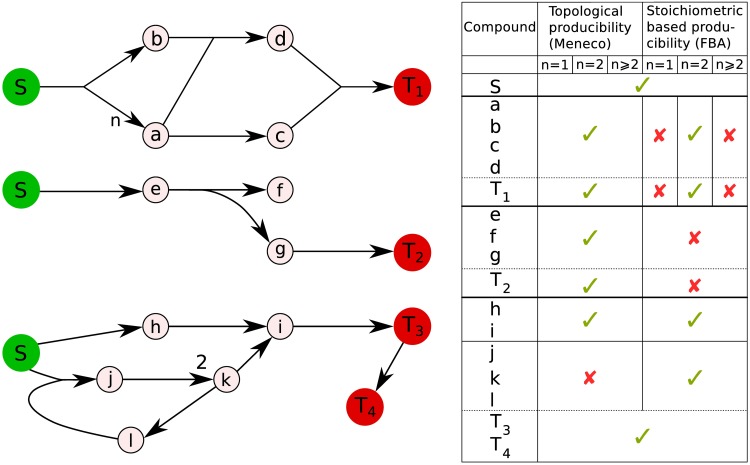
Topology and stoichiometry-based producibility of compounds in a metabolic network. Seeds and targets are *S* and *T* circles, respectively. The objective functions are formed by a reactions consuming the ensembles {*T*_1_}, {*T*_2_} and {*T*_3_, *T*_4_}. Arrows represent reactions. The labels of the reactions *S* ↦ *na* + *b* and *J* ↦ 2*k* depict their stoichiometry. Crosses indicate that metabolites cannot be produced. Check marks indicate that metabolites can be produced. The compound *T*_1_ can always be produced according to graph-based criteria whereas the variation of the stoichiometric coefficient *n* can block the production of *T*_1_ according to a balanced-mass stoichiometric framework: Flux Balance Analysis (FBA). By blocking the production of *T*_1_, a variation of *n* can also block the production of all metabolites downstream. The compound *T*_2_ can be produced according to graph-based criteria whereas the fact that *f* cannot be accumulated blocks the production of *T*_2_ according to a balanced-mass stoichiometric framework. On the other hand, *k* remains FBA-producible through the cycle involving *j*, *k* and *l* whereas it is not producible according to our graph-based criteria. *T*_3_ and *T*_4_ are producible by both criteria.

We designed the Meneco tool, which uses qualitative constraints to express the producibility of metabolites purely based on the topology of the metabolic network. Following a qualitative approach to elaborate the bio-synthetic capacities of metabolic networks according to [25–27], Meneco defines the synthetic capability of the system, with respect to a set of input compounds, as the set of metabolites that can be produced following a route in the network. As described in the third part of Fig 2, this definition excludes the self-production of a compound via cycles as it is allowed in mass-balanced stoichiometric frameworks: Meneco requires the independent production of all inputs in the cycle to “initiate” the reactions [28].

In the first part of Fig 2, we specifically illustrate the impact of stoichiometric coefficients on different definitions of producibility. In this example, all the metabolites would be producible from the seed *S* using a topological approach. In contrast, using a FBA-based approach, the stoichiometric coefficient *n* will have a great influence on the producibility. The first criterion to produce *T*_1_ is to have an equal quantity of *d* and *c* available. One *a* will be used to produce one *d* and a second *a* is necessary to produce one *c*. Consequently, the only way to produce *T*_1_ using the stoichiometric approach is to have the coefficient *n* equal to twice the value of the stoichiometric coefficient of *b*, here 2. It is also interesting to note that, if the objective function was only formed by the reaction producing metabolite *d*, the only way to enable it from a stoichiometric point of view would be to have *n* = 1. Using a topologic based approach, *d* would be always producible from *S*.

Based on this topological producibility criterion, Meneco uses the ASP paradigm [29] to efficiently model the logic of bio-synthetic producibility and to solve the gap-filling problem as a combinatorial optimization problem. For each network that does not satisfy this criterion, Meneco attempts to complete the network by importing minimal sets of reactions from a metabolic reference database such that the resulting network provides the required functionality with respect to topological metabolite producibility. The ASP technology is not only able to enumerate all minimal network completions, but also to compute the union and intersection of all minimal network completions without enumerating them, leading to strong gains in efficiency while still being exhaustive. Applied to the example depicted in Fig 1, the five different sets of reactions restoring the topology-based producibility of *T*_1_, *T*_2_ and *T*_3_ are: {*R*_3_, *R*_4_, *R*_7_}, {*R*_3_, *R*_6_, *R*_7_}, {*R*_4_, *R*_5_, *R*_7_}, {*R*_6_, *R*_7_, *R*_8_}, {*R*_4_, *R*_7_, *R*_8_}. Meneco missed the reactions *R*_1_ and *R*_2_, so did GapFill, because of the parsimonious criteria they used. On the contrary, Meneco reported the reactions *R*_5_ and *R*_8_ because it checked that *T*_2_ could be simply produced by adding *R*_8_ as soon as *T*_1_ was produced. Finally due to the cycle, Meneco missed the reaction *R*_9_. This could have been circumvented by adding one of the metabolites of this cycle to the seeds, like explained in the section dedicated to the application of Meneco to *E. mutabilis*. In particular, as Meneco is a graph-based approach, it may fail to capture the intrinsic non-linearity of metabolic behaviours [30] as well as flux imbalances [31]. The other tools, as they rely on the stoichiometry of metabolic reactions, are more relevant for the study of metabolic networks regarding such properties.

#### Benchmarking gap-filling methods

As described in the introduction, the main application area of Meneco is the reconstruction of GEMs for non-classical organisms. The automatically reconstructed bacterial GEMs in the BioCyc repository [17] contained 8% fewer reactions than the curated bacterial GEMs. Based on this observation, we generated a large-scale benchmark of 3,600 degraded GEMs as follows. Ninety biomass functions for the *iJR*904 *E. coli* GEM [32] were randomly generated. Then, forty GEMs were obtained by removing 10%, 20%, 30%, or 40% of the same *iJR*904 *E. coli* GEM. Degradation occurred through all types of pathways, including the central ones, such as the Tri-Carboxylic Acid cycle (TCA) (See Section 2 in S1 Appendix). Reactions removed from *E. coli* network *iJR*904 were classified according to their functionality with respect to biomass production. A reaction *r* was defined as essential when a non-zero biomass production implied a non-zero flux through this reaction. For instance, the reaction *R*_7_ is essential for the production of *T*_1_ in the network depicted in Fig 1. Otherwise, if a pathway can produce the biomass without involving reaction *r*, then the latter is considered alternative. This is the case for reactions *R*_3_ and *R*_5_ with respect to the production of *T*_2_ in Fig 1. Finally, if the flux through reaction *r* is always zero, this reaction was classified as blocked. Please note that the classification of reactions into essential, alternative and blocked in this article is related to and highly dependent on the corresponding biomass reaction. Importantly, the degradation of *E. coli* networks carried out in our benchmarks was such that essential, alternative, and blocked reactions, with respect to each of the 90 different biomass functions, were uniformly removed from the initial network. Together, we obtained a benchmark of 3,600 (40*90) GEMs. None was capable of producing the corresponding biomass.

In order to complete these degraded metabolic networks, we selected the MetaCyc reference database (version 18.5) [17], motivated by both its wide content (eukaryotic and prokaryotic reactions) and its accessibility (freely downloadable). This benchmark represents the use-case where the networks that must be gap-filled depict organisms that cannot be naturally associated to a precise phylogenetic taxa (see for example the case of *E. siliculosus*), requiring to explore all possible metabolic reactions to fill the network. The identifiers of *iJR*904 were manually mapped to identifiers of the MetaCyc database and both files were merged to create a complete reference database which contains all the reactions removed from the original GEM.

#### Meneco and fastGapFill efficiently complete GEMs using reference database of realistic size

The effect of the parsimonious criterion of Meneco, coupled with the fact that stoichoimetry information is not taken into account, can be seen in the benchmark in S1 Appendix. In this benchmark we completed 10,800 degraded *E. coli* networks with the MetaCyc database. The completion was performed using either the fastGapFill, MIRAGE or Meneco tools. Even if this comparison is not entirely representative due to the different backgrounds and aims of the methods, it is worth noting that Meneco in general selected smaller sets of reactions to complete the networks compared to the other methods. Indeed, on average, Meneco returned answers 8 times smaller than fastGapFill and 125 times smaller than MIRAGE. This could enable an easier and faster manual curation of the results. Nevertheless this greatly reduced number of reactions proposed comes with a cost: Meneco restores less functionality, especially when it comes to highly degraded networks. The results of this benchmark are summarized in Table 1. The large number of enumerated solutions to the topological parsimonious problem confirmed that GapFill could not be used to perform an exhaustive gap-filling of the GEMs in practice. Moreover the analysis suggests that MIRAGE is not suited to be used with minimal data (draft network, seeds, targets and no *a priori* scoring on the database) for gap-filling and that the algorithm needs all the recommended data (phylogenetic and/or transcriptomic scores) to perform correctly and gain robustness.

**Table 1:** Size of the completions and capability to produce biomass of GEMs in the benchmark of 3,600 degraded *E. coli* GEMs after their completion with Meneco, fastGapFill and MIRAGE. Between 10% and 40% of reactions were removed from *E. coli* *iJR*904 reference network in order to block the FBA-based production of 40 different biomass functions. For each degraded GEM, both the Meneco and fastGapFill tools were used to restore the producibility of the biomass by picking reactions from the MetaCyc database. For 10% of the degraded GEMs in the benchmark, the MIRAGE tool was also tested.

Degradation rate of GEMs in the tested benchmark					
	All	10%	20%	30%	40%
**Removed reactions**					
min	101	101	203	307	414
max	446	117	226	343	446
mean	270	109	216	325	430
**Reactions added by** **Meneco**					
min	0	0	4	6	13
max	110	23	36	62	110
mean	32	10	22	38	59
**Reactions added by** **fastGapFill**					
min	150	150	213	284	339
max	388	209	256	350	388
mean	273	180	237	311	366
**Reactions added by** **MIRAGE**					
min	3481	3517	3678	3687	3481
max	4228	3951	4048	4145	4228
mean	4029	3916	4005	4094	4131
**Percentage of filled GEMs able to synthesize biomass**					
Meneco	40.83%	73%	36%	35%	19%
fastGapFill	72.88%	92%	92%	50%	58%
MIRAGE	100%	100%	100%	100%	100%

#### Meneco improves the accuracy of the reactions added to filled GEMs compared to fastGapFill

**Fig 3:**
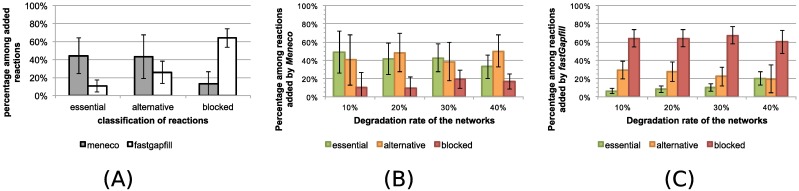
Classification of reactions added by Meneco and fastGapFill in functional completed GEMs. For each degraded GEM which recovered its ability to synthesize biomass after gap-filling, the functional classification (essential, alternative, blocked with respect to to biomass production) of reactions added to the GEM was calculated. (A) Comparison of Meneco and fastGapFill over the complete benchmark in terms of biomass production restoration. (B). Classification of reactions added in functional GEMs filled by Meneco. (C). Classification of reactions added in functional GEMs filled by fastGapFill.

In order to estimate the accuracy of Meneco and fastGapFill with regard to the proposition of reactions to produce targets, all reactions added to a functional GEM for gap-filling were classified into essential, blocked and alternative reactions with respect to the production of biomass. The analyses are depicted in Fig 3. On average, 63% of the reactions added by fastGapFill were blocked in the completed GEM towards the production of the biomass, *i.e.* the production of the target compounds. This high rate of blocked reactions was independent of the initial rate of degradation of the considered GEM. This is consistent with the main purpose of fastGapFill which aims at unblocking all reactions in the GEM core set without any focus on a set of targeted reactions. Conversely, only 12% of reactions added by Meneco were blocked with respect to the biomass reaction in the completed GEMs, and even less for 10% and 20% degraded GEMs. This confirms that fastGapFill does not solve the exact same problem as Meneco does, that is proposing sets of reactions to produce unproducible compounds. Since the problem fastGapFill solves is a larger one, it is more difficult to filter and manually curate the proposed solution as it is often required after gap-filling. Meneco is a relevant tool for the preliminary completion of a new draft metabolic network when limited phenotypic information are available. Its parsimonious feature allows suggesting reactions directly related to the production of the targets and manual curation is facilitated through a study of the whole space of solutions.

### Functionality analysis: Meneco recovers essential reactions of a metabolic network

Our results suggest that the topological parsimony criterion used in Meneco provides a good trade-off in terms of scalability with respect to the size of the reference database used for completion: the output of Meneco remains reasonable in terms of size (for an *a posteriori* manual curation), with a reasonable loss of impact on the restoration of biomass production compared to parsimonious approaches.

To further evaluate the quality of the output of Meneco, we completed the 3,600 degraded GEMs of our benchmark derived from the *iJ*904 *E. coli* metabolic network by 7,200 (2*3,600) additional degraded GEMs of the *iAF*1260 [32] and *iJO*1366 [33] *E. coli* networks. The Meneco tool was applied to complete each of the 10,800 degraded GEMs by using the networks prior to degradation as a reference database. The main motivation for changing the reference database was to be able to analyze how the classification of reactions into essential, alternative and blocked with respect to biomass production evolved with the completion process.

#### Impact of the gap-filled network and the reference database

**Fig 4:**
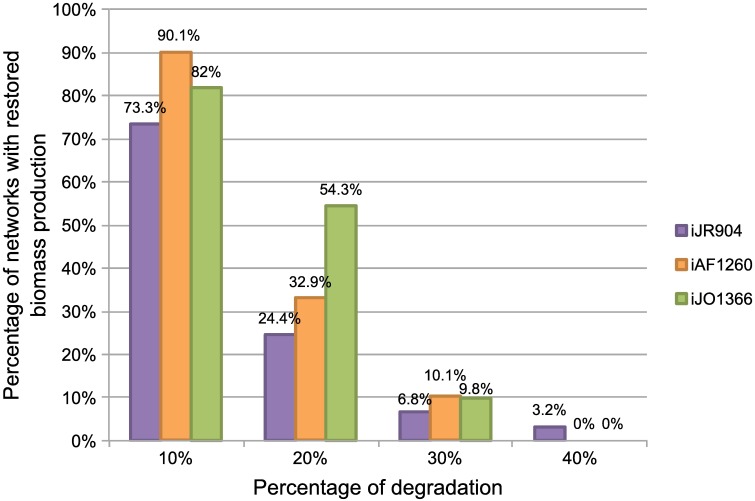
Applying the gap-filling procedure Meneco on three benchmarks built from *E. coli* networks of different quality. For three different reference networks (*iJR*904, *iAF*1260 and *iJO*1366), the Meneco tool was applied to 3,600 pairs consisting of a degraded *E. coli* metabolic networks (40 different networks with levels of degradation indicated by the abscissa) and a random biomass reaction (90 different targets sets). The initial *E. coli* network was used as a reference database to perform the completion. The percentage of functional networks after completion is indicated on the ordinate axis.

The results of the completion procedure detailed in Sections 2 and 5 in S1 Appendix show that the quality of the network used to generate the benchmark has a low impact on the size of the Meneco output, even if the size of the solution set increased linearly with the degradation rate of the network. Nevertheless, the quality of the reference *E. coli* network has a strong impact on the quality of the results as shown in Fig 4. Similarly, we noticed that the reference database used for gap-filling has a strong impact of the results: adding the MetaCyc database to the original *E. Coli* network multiplied by 6 the percentage of functional networks after completion (see Sections 2 and 5 in S1 Appendix).

#### Identification of essential and alternative reactions with Meneco

The reactions of the 40 degraded i*JR*904 were classified according to their functionality with respect to the production of their associated 90 random biomass functions. According to this classification, we tested how many essential, blocked and alternative reactions (with respect to the biomass production) were recovered in the networks filled by the Meneco procedure. Our analysis shows that Meneco was able to recover 97.5% of the essential reactions on average among the 10,800 experiments, and few blocked reactions were included in the networks filled by this tool.

**Fig 5:**
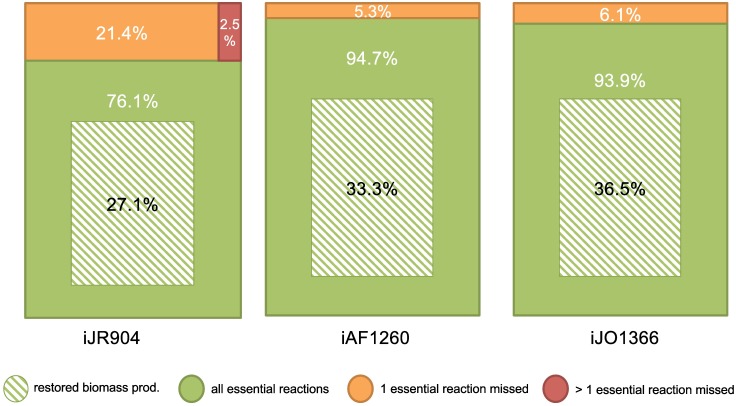
Biomass restoration and recovery of essential reactions due to completion of 10,800 degraded networks by Meneco. For the 10,800 degraded *iJR*904, *iAF*1260 and *iJO*1366 networks, the gap-filling results were classified according to their status: (i) restored biomass production (green and white stripes), (ii) recovery of all essential reactions (green), (iii) exactly one missed essential reaction (orange) and (iv) more than one missed essential reaction (red).

To gain better insights into the importance of essential and alternative reactions in the gap-filling procedure, we classified each gap-filling experiment according to four categories: (i) the network is functional after gap-filling (ii) the network has recovered all essential reactions after gap-filling but it is not functional (iii) the gap-filling procedure missed one essential reaction and (iv) the gap-filling procedure missed more than one essential reaction. Results are depicted in Fig 5. They confirm that in 9,529 completions (88.2%), Meneco recovered all essential reactions of the reference network. When failing to restore network functionality, it still recovered all essential reactions in 6,041/7,312 completions (82.3%). In the cases when Meneco did not recover all essential reaction, it generally missed a single essential reaction, and at most 3 (in only 90/10,800 cases—0.8%).

Failure to restore biomass production in FBA is mainly explained by missing alternative pathways which were not restored by the gap-filling procedure. This was confirmed by analyzing the status of reactions in the 3,488 networks reconstructed with Meneco and capable of producing biomass: among the reactions that were essential in the reconstructed network, on average 40% were classified as alternative in the reference network (see S1 Files). Similarly, 47% of the blocked reactions in gap-filled networks were classified as alternative in the initial one.

#### Parsimonious topological gap-filling conserves the role of reactions

We further investigated the networks after their gap-filling by Meneco and compared them to their reference network, to check whether the gap-filling process changed the classification of reactions. As Flux Variability Analysis (FVA) can only be applied if biomass can be quantitatively produced, we studied the 3,488 networks (among the 10,800) capable of biomass production after their gap-filling. To do so, we compared the classification of reactions before degradation to the classification obtained after degradation and gap-filling. In all cases, reactions that were initially blocked with respect to biomass production remained blocked after the completion. In the same way, 98.6% of the reactions initially essential remained essential after the completion, the others becoming alternative (see S1 Appendix). This slight difference could be explained by rounding errors made by the solver when computing FVA. The study of reactions that were initially alternative is most interesting. Since the completion by Meneco is parsimonious, one can expect some of the initially alternative reactions to become either blocked or essential, by blocking some long production pathways and making the shortest one essential for biomass production. Examples of such situations are depicted in Section 3 in S1 Appendix. 63.3% of the reactions remained alternative, while 34.7% became blocked and 2% essential. On average, for each reconstructed network, 30 initially alternative reactions became essential. From our point of view, this number is low enough to enable a manual curation of the completion results, once more demonstrating that Meneco is a good decision support tool. It is also interesting to note that the quantity of reactions that change classification is similar for each of the three tested networks.

Meneco restores the essential and blocked features of the reactions according to the available information. Due to the minimality criterion, it prefers shorter pathways, thus turning some alternative reactions into either blocked or essential reactions. A change from essential to blocked for a reaction would indicate a failure in the gap-filling process. Since an essential reaction is mandatory to restore biomass production in FBA, gap-filling methods always retrieve functional pathways retaining reactions that were initially essential. While keeping the essential characteristic of the reactions is important, the fact that blocked reactions remain blocked is a sign that performing the gap-filling did not create new functional pathways.

#### Topological parsimonious gap-filling outperforms stoichiometric-based gap-filling on degraded GEMs

Previous experiments show the added-value of parsimony towards the selection of adequate gap-filling solutions. As a final study, all the analyses were run with the GapFill method to estimate the impact of the choice of the producibility criterion (topological vs stoichiometric) over the parsimonious gap-filling procedure. The GapFill benchmark was run as single target completion since it was impossible to be run on a global biomass function while being exhaustive. This is due to the combinatorial characteristics of the problem: the number of solutions when filling the networks based on a complete biomass was too high (mandatory bounding of the number of solutions with GapFill) and so was the computation time. Hence, we used as output for GapFill the union of the completion sets (maximal 30) for each individual target. We noticed that only 68 networks reached the limit of 30 completion sets for individual targets among the 10,800 degraded networks.

Interestingly, although Meneco and GapFill solutions are comparable in sizes, they only share 45.3% of the reactions (see Section 4 in S1 Appendix). On average, GapFill returned 1.6% more reactions (*i.e.* 4) than Meneco. Ability to restore biomass production (FBA) was lower for GapFill than for Meneco: 21.6% and 32.3% respectively on 10,800 networks, and particularly for 10% degraded networks: 49.4% and 81.9% respectively. Recovery of essential reactions was less efficient for GapFill: it missed more than one (and up to 21) essential reactions in 13.9% of the cases, a major contrast with Meneco (0.8% and no more than 3 missed reactions). Analyses and results are available in Section 6 in S1 Appendix.

Altogether these features confirm that the topological over-approximation, when compared to stoichiometric criteria, is relevant for the parsimonious gap-filling of a new draft metabolic network not only in terms of performances but also in terms of functional accuracy.

### First application: The Meneco tool may help to elucidate the respective roles of species in a symbiotic community

#### Comparing the production of targets when merging two networks

The Meneco tool was applied to the analysis of candidate algal-bacterial interactions in the brown algal model *E. siliculosus*. Interactions between algae and their associated bacteria are of increasing interest because of the potential key roles bacteria may have on the biology of the algae, in particular through metabolic connections between both types of organisms [22]. For this purpose, we considered, as a fixed set of seeds, molecules found in the seawater medium and commonly used to grow these organisms [21]. In parallel, we designed a large set of targets by identifying all reactions in MetaCyc 19.0 for which at least one of the associated genes was supported by the presence of one or more expressed sequence tag (EST) within the dataset of 90,637 ESTs published in the original *Ectocarpus* genome paper [34]. For each of these expressed reactions, we considered that both reactants and products should be available in the network and defined them as targets. Using Meneco we then identified which of these targets were producible in two situations. Firstly, we used as a draft network the algal network itself, more precisely a version of EctoGEM 1.0 [21] for which no functional gap-filling had been carried out. Secondly, we used as a draft network the union of the aforementioned algal network and of the metabolic network of *Candidatus* Phaeomarinobacter ectocarpi, a bacterium suggested to live in association with *E. siliculosus* [20].

**Fig 6:**
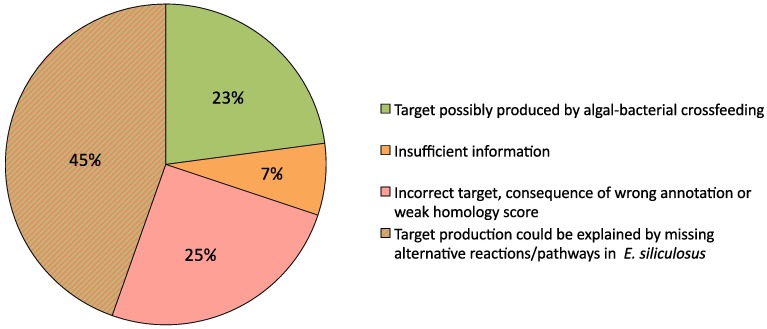
Study of the 83 newly producible targets when combining *E. siliculosus* and *Ca.* P. ectocarpi metabolic networks. When merging *E. siliculosus* and *Ca.* P. ectocarpi metabolic networks, 83 targets previously non-producible by *E. siliculosus* became producible. The 93 essential reactions related to their production by the *Ca.* P. ectocarpi network as well as the *E. siliculosus* ESTs were studied to assess whether the new producibility may be the result of a real interaction between the two organisms.

A comparison of both cases allowed the identification of targets that became producible when adding the metabolic capacities of the *Ca.* P. ectocarpi network to the algal network. These targets and the metabolic pathways enabling their production were then manually examined. In total 83 previously non-producible algal targets became producible according to the Meneco semantics when combining the metabolic capacities of the bacterium with those of the alga. For each of these 83 targets, the essential reactions allowing its producibility by *Ca.* P. ectocarpi were studied, as well as the target and its related EST in *E. siliculosus*. The purpose was to determine whether the production of the target by the alga could depend on an interaction with the bacterium. These individual examinations revealed some errors or missing annotations in *E. siliculosus* as well as some cases of weak sequence homology leading us to no longer consider 21 (25%) of the 83 compounds as targets. Improvements in genome annotations also allowed recovering alternative reactions or pathways in *E. siliculosus* related to the production of 37 (45%) of the compounds. Nineteen compounds (23%) however are likely to be part of an exchange between the alga and the bacterium (Fig 6). The production of a target was considered the result of a possible interaction between the two species if i) the algal EST related to the target definition was correctly annotated (*i.e.* confirmation of the target), ii) the target was not producible by the algal itself through the presence of other ESTs or genes and iii) the bacterial reaction(s) necessary for the target production was (were) correctly annotated. Details are available in S2 Files.

#### Identifying candidate pathways for algal-bacterial interactions

Several of the reactions identified by Meneco were the result of potentially interesting interactions.

For example, *E. siliculosus* on its own is probably incapable of producing histidine or histidinol because a histidinol-phosphatase (EC 3.1.3.15) is missing from its genome. Meneco has previously identified this mandatory reaction to complete the histidine biosynthetic pathway [21]. Recent genomic data from other strains of *E. siliculosus* (Dittami, Tonon, personal data), as well as the recently published genome of *Saccharina japonica* [35], confirm the absence of a histidinol-phosphatase in other brown algae, while a corresponding enzyme is present in diatoms. As a proteinogenic amino acid, histidine is essential for all living organisms. Brown algae have therefore either evolved an alternative way of producing histidinol or histidine, or they acquire at least one of these substances from their environment. Our analyses indicate that histidinol or histidine may be provided by symbiotic bacteria such as *Ca.* P. ectocarpi, which encodes all enzymes of the histidine biosynthetic pathway.

**Fig 7:**
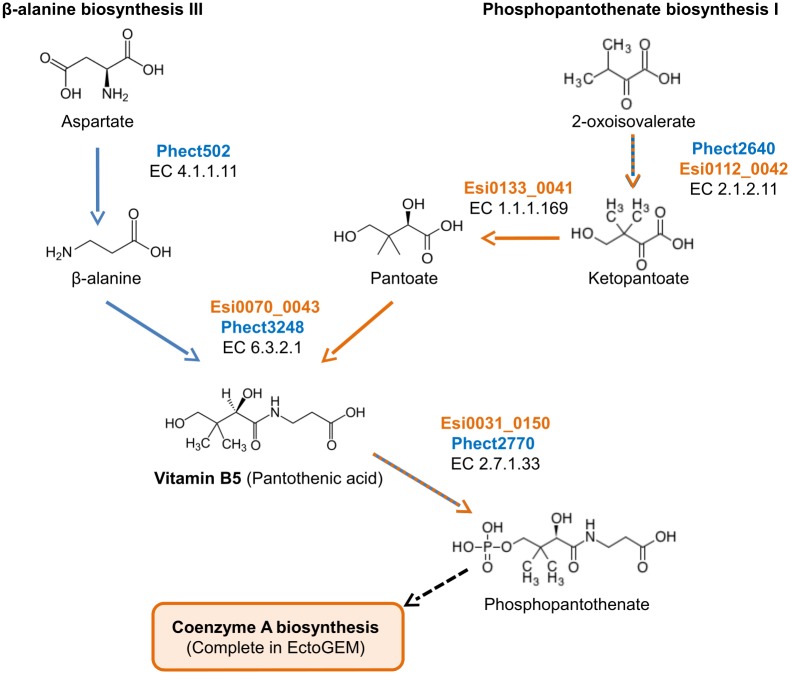
Vitamin B5 biosynthesis in *E. siliculosus* and *Ca.* P. ectocarpi. Orange labels designate enzymes from the alga, blue labels correspond to enzymes from the bacterium.

A second example is vitamin B5 (pantothenic acid), which is an important vitamin for the formation of coenzyme-A. *E. siliculosus* is capable of producing vitamin B5 from *β*-alanine via the activity of a pantoate-*β*-alanine ligase (Esi0070_0043), but it lacks a biosynthetic pathway to produce *β*-alanine (Fig 7). Our analysis suggests that *E. siliculosus* may rely on external sources of either *β*-alanine or vitamin B5. The bacterium *Ca.* P. ectocarpi is able to provide both of these compounds via the phosphopantothenate biosynthesis pathway.

A third example is agmatine, an aminoguanidine, which is well-studied in human where it modulates for instance receptors of neurotransmitters or ion channels. While the role of agmatine in *Ectocarpus* still remains unknown, the *E. siliculosus* metabolic network possesses two metabolic reactions (MetaCyc ID: AGMATINE-DEIMINASE-RXN, E.C. 3.5.3.12 and MetaCyc ID: AGMATIN-RXN, E.C. 3.5.3.11) for the degradation of agmatine and polyamine synthesis. These reactions in *Ectocarpus* are well-supported, despite the fact that it lacks the metabolic capacity to produce agmatine, as no arginine decarboxylase (E.C. 4.1.1.19) is present in its genome. As in the case of histidine synthesis described above, this latter observation also holds true for other sequenced *Ectocarpus* strains and the *S. japonica* genome. A plausible explanation is that *E. siliculosus* (and other brown algae) may use external sources of agmatine to produce polyamines. *Ca.* P. ectocarpi is capable of producing agmatine, which constitutes a potential interaction point between both organisms. Our current working hypothesis is that when external or bacterial agmatine is available, agmatine-derived polyamines may complement or replace ornithine-based polyamine synthesis in *E. siliculosus*.

These three examples provided interesting working hypotheses that will now need to be tested experimentally. Corresponding experiments are ongoing but time-consuming as they first require separate cultivation of algae and bacteria.

#### False positive interactions

The aforementioned three examples highlight promising candidate pathways for algal-bacterial interactions. For other identified metabolites and reactions, however, sequence information was insufficient to determine the exact specificity of the enzymes and reactions, and thus to draw conclusions without additional experimentation. Furthermore, in S2 Files and Fig 6, we analyzed the 58 targets leading to false positive results (70%), i.e. candidate algal-bacterial interactions either due to missing gene annotations or false/overly precise annotations. One example for each of these cases is detailed in S2 Files: one missing annotation in the algal genome falsely leading the assumption that the bacterium was needed to provide riboflavin, and one wrong assignment of EC numbers to an algal gene leading to predict a reaction involved in peptidoglycan synthesis.

### Second application: The Meneco tool enabled a functional metabolic network for *E. mutabilis* to be built based on metabolomic and *de novo* transcriptomic data

As a second case study, we focused on the reconstruction of a metabolic network for *E. mutabilis*, a photosynthetic protist and primary producer in acidic aquatic environments. Despite important roles of *E. mutabilis* in those ecosystems and despite the fact that it has often been considered as an indicator species for acid mine drainages, this organism has been poorly described so far.

Due to their complexity and size, no genome sequences are available so far within the genus *Euglena*. Therefore, we used assembled transcript sequences as described in [18] and annotated them by similarity search against enzyme reference sequences from MetaCyc. We used a stringent similarity threshold for function assignation to reduce as much as possible the rate of false positive reactions included in the initial network that was subsequently completed by Meneco. This ensured that the reconstruction of the *E. mutabilis* metabolic network would be highly conservative, avoiding the inclusion of reactions based on false annotations. The major drawback of this approach was the resulting high rate of missing reactions in the initial draft network, which did not allow Meneco to restore the production of all targets at once. The 72 targets are listed in Supplementary files S6. Of those targets, 54 were defined according to [19] who showed that they were secreted or accumulated in *E. mutabilis* cultured in a minimal mineral medium. The 17 other targets were myristic acid, shown to be present in the *E. mutabilis* membrane [36], and the remaining proteinogenic amino acids, nucleotides, and chlorophyll A and B.

Running Meneco with the 8 mineral seeds and |Light| (Supplementary files S6) could propose a completion solution only for UREA and OXYGEN-MOLECULE among the 72 targets. These two metabolites could be produced only because they were the products of a single reaction involving only mineral reactants, a situation which was not biologically satisfying.

#### Adding the appropriate seeds to unblock the draft network

The principal reason for the near impossibility to produce the targets lies in the fact that all targets and internal metabolites of the *E. mutabilis* metabolic network must be produced from the mineral seeds only, including cofactors that are essential to the vast majority of reactions. These cofactors themselves depend for their biosynthesis on metabolites that they contribute to produce. For instance, NAD is biosynthesized from aspartic acid or tryptophan, two amino-acids whose production requires NAD. In other words, most of the *E. mutabilis* initial draft network consisted of a complex cycle with long range circular dependencies that no external mineral nutrient could initiate. Thus, this network could not produce the targets with Meneco without adding the appropriate extra seeds to solve the circular dependencies. The addition of NAD to the seeds permitted to initiate the draft network, enabling Meneco to restore the production of 69 targets out of 72. The addition of |ACP| to the seeds was required to restore the production of two remaining targets—PALMITATE, CPD-7836 (myristate) –, and adding |Red-Thioredoxin| to the seeds was necessary to restore the production of DCTP (deoxycytidine-triphosphate).

#### Solving local cycles and circular dependencies

Although the production of all the targets was enabled by the addition of the 3 seed metabolites NAD, |ACP| and |Red-Thioredoxin|, some pathways could still be non-functional with Meneco because of local cycles and circular dependencies. For instance, in oxygenic photosynthesis, oxygen is produced by photosystem II: light provides the energy for transferring 4 electrons to 2 molecules of plastoquinone from 2 molecules of water which are split into 4 protons and one molecule of oxygen. Since the |PLASTOQUINONE| compound was not available in the network, Meneco could not return the correct reaction PSII-RXN as a solution for the production of OXYGEN-MOLECULE, but proposed instead to reverse the reaction of water-forming NAD oxidation RXN-14692.

Since *E. mutabilis* is a primary producer, the photosynthesis reactions are at the origin of biomass production. The photosynthesis pathway can be divided into two subpathways: the light reactions collecting the energy from light and the Calvin-Benson-Bassham cycle, also known as the dark reactions, building the precursor components of biomass from carbon dioxide.

**Fig 8:**
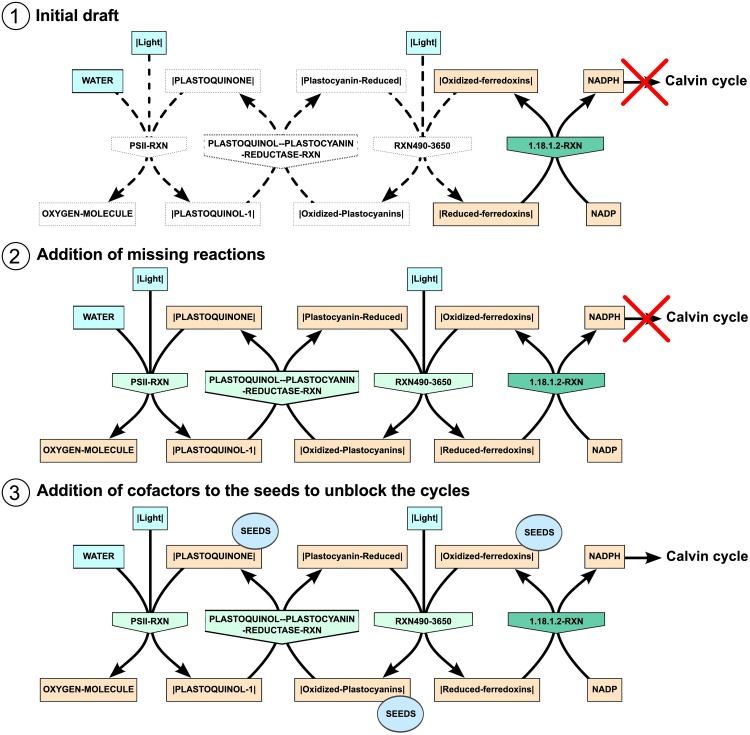
Photosynthesis light reactions. This pathway is a chain of oxidoreduction reactions transferring electrons from water to NADPH which will be used as a reductor in the Calvin-Benson-Bassham cycle. It is equivalent to a succession of three cycles of two reactions each and as such is not functional for Meneco unless the appropriate seed metabolites are added. The metabolites in light blue are those corresponding to the actual nutrients in the original set of seeds. The three metabolites that had to be included in the seeds to unblock each of the successive cycles are marked with a blue circle. If these metabolites are absent from the seeds, Meneco cannot propose a solution including the first three reactions, in light green, that were missing in the initial draft. Since NADP can be synthesized from NAD, there can be a functional path leading from WATER to NADPH.

Before addressing the capacity of the Calvin cycle to produce biomass precursors, we manually completed the light pathway by adding the missing reactions (PSII-RXN, PLASTOQUINOL–PLASTOCYANIN-REDUCTASE-RXN, RXN490-3650) and checked whether they could provide a viable route in the draft network (Fig 8). This pathway is actually a chain of electron transfers involving a series of donors and acceptors recycled at each step, ending with the production of the NADPH which enters the Calvin-Benson-Bassham cycle. Each pair of successive reactions thus constitutes a small cycle which needs to be solved in order to make the whole chain functional and to enable the production of NADPH. Except for NADPH/NADP, none of the concerned donor/acceptor couples have any *de novo* biosynthesis pathway described in MetaCyc, and therefore, the production of the corresponding donor and acceptor are mutually dependent. In order to unblock the whole chain, we added the electron acceptors (|PLASTOQUINONE|, |Oxidized-Plastocyanins| and |Oxidized-ferredoxins|) to the seeds, with the exception of NADP which could be produced from NAD as shown in Fig 8.

To verify that the chain of photosynthetic light reactions was functional, we isolated the individual reactions (PSII-RXN, PLASTOQUINOL–PLASTOCYANIN-REDUCTASE-RXN, RXN490-3650, 1.18.1.2-RXN) in a subnetwork and checked for the production of NADPH with Meneco using the same seed metabolites as for the whole network. A single solution was returned with three reactions, NADPYROPHOSPHAT-RXN, APYRASE-RXN, NAD-KIN-RXN, producing respectively AMP from NAD, ATP from AMP, and eventually NADP from NAD and ATP. This confirmed that the four reactions of the photosynthesis light pathway were able to produce NADPH provided that NADP was available.

The same dependence on ATP was observed with the whole draft network which could already produce AMP but not NADP although the reaction NAD-KIN-RXN of NAD phosphorylation into NADP was present. Running Meneco on the whole draft network to check its capacity to produce NADPH returned two solutions of one reaction each, RXN-10862 and APYRASE-RXN, both allowing the production of ATP from AMP and unblocking the reaction NAD-KIN-RXN. At this stage, ATP was provisionally added to the seeds until more information could be gathered about which reactions should be included in the network for its production, and to enable the photosynthesis pathway to provide the Calvin-Benson-Bassham cycle with the necessary NADPH.

The Calvin-Benson-Bassham cycle is the pathway of the photosynthesis process that reduces carbon dioxide to produce glucides. The net equation of this cycle can be written as:

$$3\;{\text{CO}}_{2}+9\text{ ATP}+6\text{ NADPH}+6\;{\text{H}}^{+}\to {\text{C}}_{3}{\text{H}}_{4}{\text{O}}_{7}\text{P}+9\text{ ADP}+8\;{\text{P}}_{i}+6\;{\text{NAD}}^{+}$$

The net production of 3-phospho-glyceric acid (G3P) is the main source of organic carbons for further biosynthetic processes, but other metabolites may exit the cycle towards glycogenesis, amino-acid-, nucleotide-, or lipid biosynthesis. In order to assess whether the Calvin-Benson-Bassham cycle was functional we therefore tested the draft network for the production of G3P, FRUCTOSE-6P, RIBULOSE-5P, GAP and DIHYDROXY-ACETONE-PHOSPHATE. Three solutions were proposed by Meneco with one reaction each: AMP-DEAMINASE-RXN, AMP-NUCLEOSID-RXN and NMNNUCLEOSID-RXN. The latter two reactions directly produce |RIBOSE-5P| whereas the first one yields IMP which can be used to produce |RIBOSE-5P| as well, suggesting that the Calvin-Benson-Bassham cycle requires this metabolite to be functional. We thus added |RIBOSE-5P| to the seeds and all 5 targets previously tested with the whole network were now producible by a subnetwork composed of only the 13 reactions of the Calvin cycle, confirming that this pathway was now functional.

It is worth noting that, at this stage, the metabolite seed |RIBOSE-5P| could be replaced by D-RIBULOSE-15-P2. This solution was provisionally preferred as this compound is a reactant of the carbon fixation reaction in photosynthesis. Thus, adding it to the seeds, along with ATP, may simulate the initial batch of metabolites that allows *E. mutabilis* to initiate the Calvin-Benson-Bassham cycle.

The photosynthesis pathway being functional, a first route was now open between the mineral nutrients (seed metabolites) and the internal and excreted metabolites. Carbon-based biomass precursors such as G3P or FRUCTOSE-6P were producible and could enter the central metabolism. The next step was therefore to address the incorporation of nitrogen, in the form of ammonium (NH_3_), in order to trigger the metabolism of amino-acids and nucleotides. Since the Tri-Carboxylic Acid cycle (TCA) is at the intersection between the metabolisms of glucides, lipids and amino-acids, this pathway was completed manually to ensure that it could use the G3P coming from photosynthesis. The following 5 reactions were thus added to the draft network: MALATE-DEHYDROGENASE-ACCEPTOR-RXN, ACONITATEDEHYDR-RXN, 2OXOGLUTARATEDEH-RXN, ISOCIT-CLEAV-RXN, PYRUVDEH-RXN. Furthermore, CO-A was provisionally added to the seeds as the TCA depends on ACETYL-COA. Assessing with Meneco whether the TCA was able to produce MAL (malate), CIT (citrate) or 2-KETOGLUTARATE suggested that either GLYOX (glyoxylate) or OXALACETIC_ACID should be added to the seeds in order to unblock the TCA cycle. Both compounds are involved in many reactions in MetaCyc, however, as the addition of OXALACETIC_ACID to the seeds would have artificially enabled the production of ATP, GLYOX was used as a seed instead.

Checking the current draft with Meneco for the production of amino-acids, 5 were already producible (GLT, L-ASPARTATE, GLN, L-ALPHA-ALANINE and GLY). Since glutamic acid (GLT) is the principal component resulting from the incorporation of ammonium and a key metabolite in nitrogen metabolism, we had thus achieved the completion of the metabolic backbone leading from mineral nutrients through photosynthesis to carbon and nitrogen metabolism.

The same process was repeated successively for the targets listed in Supplementary files S6 until all were producible. After the addition of new reactions, the provisional seeds were removed to test if they were still needed or if a biologically satisfying completion could replace them.

#### Test of the completed draft network with FBA

Once all the targets could be produced by the network using the Meneco criteria, we proceeded to verify whether it was functional in FBA. We created 10 import reactions (8 for mineral nutrients, one for light, and one for oxygen to enable respiration), a biomass reaction, and 54 output reactions for the 54 metabolites that were experimentally shown to be secreted by *E. mutabilis*. Forty dead-end metabolites, *i.e.* non-consumed products that may prevent the network from satisfying the stoichiometric constraints for the production of target metabolites (secreted metabolites and biomass) were identified manually and defined as boundaries (boundaryCondition=“true”). Additionally, three reactions (FLAVONADPREDUCT-RXN, RXN0-882, RXN-9944) were added to the network in order to regenerate reduced flavodoxins, oxidized ferredoxins, and thioredoxin since the corresponding transcripts could be identified in the transcriptome. We then checked which output reactions (biomass and secretion reactions) had a zero flux and determined the corresponding dead-end metabolites using FVA. Only the secretion of SPERMIDINE was thus blocked for stoichiometric reasons. We therefore added a dummy reaction of 5-METHYLTHIOADENOSINE degradation to satisfy the stoichiometric constraints on this metabolite for SPERMIDINE biosynthesis. The final draft thus contained a total of 1,068 reactions, including 10 entries, 54 secretion reactions, 1 biomass reaction and 1 additional degradation reaction. FBA showed that a positive optimum could be attained when defining the biomass reaction as an objective, and allowing only mineral nutrients and light (see S3 Files). On the contrary, no flux could be achieved in the biomass reaction if there was no entry flux for light or carbon dioxide, demonstrating that in this network the production of biomass was dependent indeed on photosynthesis. Hence, although the draft network had been completed using almost exclusively topological criteria supported by biological knowledge, the resulting network was nonetheless functional in FBA.

This model could thus provide guidelines for further exploration of *E. mutabilis* metabolism and physiology. However, the capacity of this poorly characterized *Euglena* species to feed on organic substrates, as suggested by the model, could not be demonstrated. It is not clear indeed whether it can actually grow in the dark like *E. gracilis* can, or whether it becomes dormant when photosynthesis is not possible. Laboratory experiments to determine the potential nutrient uptake of *E. mutabilis*, including *C*^13^ fluxomic to follow the fate of these nutrients, will be required to both experimentally validate and refine this model.

## Discussion

### The Meneco tool efficiently identifies essential reactions to produce large sets of targets

The main characteristic of Meneco is that it relies on a topological description of the notion of producibility. The qualitative approximation of the topological notion of producibility is more robust than stoichiometry-based notions, which are often forced to find more complete solutions that additionally fulfill the stoichiometric constraints. The logical formalization of the topological producibility allows users of Meneco to benefit from the performance of recent solvers for combinatorial problems based on ASP technologies. Meneco relies on these efficient solvers for the completion of degraded draft metabolic networks built, for instance, from NGS data. The other characteristic of the Meneco tool is that it is very flexible with respect to the definition of seeds (composition of the medium and potentially some cofactors) and targets (compounds whose producibility has to be restored). This flexibility appears to be crucial to investigate draft GEMs produced from NGS technologies, as illustrated in two applications.

### Positioning of Meneco in the landscape of gap-filling methods

Most gap-filling methods solve optimization problems over a search space whose size grows exponentially with the size of the reference database from which the reactions are taken. Two different strategies can then be used to explore the search space and compute solution sets to a gap-filling problem: either a parsimonious bottom-up strategy which enriches the draft metabolic network until the targeted properties are satisfied [8, 10, 27], or a top-down approach starting from all available information and removing reactions without added-value to the solution of the problem [15, 23, 24]. Bottom-up methods often report few solutions and may miss alternative ones while top-down approaches capture more solutions but are computationally demanding, and sometimes require sampling of the solution space. Therefore, families of gap-filling methods can be classified with respect to four characteristics: (i) the set of compounds whose producibility should be restored; (ii) the definition of producibility they rely on; (iii) the criteria they optimize; (iv) the number of solution sets they return. Meneco checks the same topological criteria for producibility as the subset-minimality top-down method [15], but it is able to enumerate all solution sets by using the parsimony criterion of the GapFill family. This allows an exhaustive computation of the family of solutions.

From a biological point of view, having a method that enables the completion of a metabolic network without the need to know the real concentration of metabolites is a real advantage. It enables a completion of the metabolic graph not only for quantified or estimated compounds, but also for those identified by qualitative measurements. Moreover, having access to an exhaustive enumeration of the possible solutions allows researchers to choose the best one among them, instead of having only a subset of solutions without knowing if this subset is representative of the entire solution space or not.

### Alternative models for topological producibility

Alternative qualitative semantics could have been used to assess the producibility of a metabolic compound. In [26, 37], Cottret *et al.* introduced refined semantics for producibility taking into account the impact of cycles on the production of metabolites. This alternative definition can be viewed as an over-approximation of FBA-consistent networks, and appears to be very useful for the efficient computation of precursor sets for metabolic targets. However, experiments consisting in encoding this alternative definition in the Meneco framework and performing gap-filling of the same 10,800 degraded networks evidenced that this alternative definition of producibility is not constrained enough to capture essential reactions. Indeed, running our benchmark on the *iJR*904 network with that definition of producibility returns a set of solutions containing on average only 59.5% of the essential reactions (S1 Files). This can be explained by self producing cycles which can occur with that definition.

### Future improvements

Our experiments using the *E. coli* benchmark evidence that, in half of the cases, Meneco (and GapFill) fail to recover enough alternative reactions to restore the biomass producibility of the network. This bottleneck can be explained by the parsimony criterion used by both tools since they identify sets with a minimal number of reactions allowing either to simultaneously restore the topological producibility of all targets (Meneco) or to enable the production of individual targets (GapFill). However, the identification of alternative routes to produce a targeted set of compounds is crucial to have a global understanding of species metabolic capability, as soon as essential reactions have been properly identified and validated. In order to improve the Meneco tool, we plan to study the impact of additional topological criteria that may take into account larger pathways. The difficulty will be to select relevant metrics in order to extend the search space while sufficiently constraining the search to be able to track the complete solution space. Indeed, with most of the methods based on extended scores and criteria for stoichiometry-based formalisms [10, 15], the space or compatible sets of reactions may become intractable, especially when many targets are considered together. In this case, most methods rely on a sampling of the solution space, which may introduce biases and errors.

Alternatively, other metrics can be developed to improve the choice of reactions by the gap-filling methods. Scores based on likelihood value computations have been introduced in [10] to improve GapFill approaches with genomic information about alternative functions for genes. In the future, it will be interesting to adapt such scores to the Meneco tool and measure their impact on the functional classification of reactions in the reconstructed network.

### Conclusion

As stated by Satish Kumar *et al.* in [8], *“clearly, the role of a gap-filling method is to simply pinpoint a number of hypotheses which need to subsequently be tested”.* In this line of research, the Meneco tool constitutes a flexible framework to pinpoint hypotheses in the context of large-scale datasets applied to newly investigated organisms.

Meneco is a versatile tool to complete draft GEMs and to suggest relevant reactions with respect to the response of the system to environmental perturbations. Importantly, it does not aim at providing a complete functional network, but rather at pointing out essential reactions and some of the alternative ones, which are crucial to explain the system response. It can be then combined with refined stoichiometry-based analyses and gap-filling methods to produce functional networks. In this sense, we promote Meneco as a tool to be used as an intermediary step within a workflow consisting of (i) producing a draft GEM [5, 38], (ii) parsimonious gap-filling based on metabolite profiles or RNA-seq datasets with Meneco, (iii) refinement of the model with stoichiometric-based approaches relying on additional data ([14] [13]), and (iv) manual curation process of metabolic networks described in [1].

From a biological point of view, the examples show that Meneco cannot replace manual curation and network analysis, but it may provide a flexible tool to aid this process. Analyses are fast, easy to implement, and invaluable because they enable biologists to focus their attention on a few highly interesting compounds and reactions without making *a priori* assumptions.

## Materials and Methods

### Topological and stoichiometric parsimonious gap-filling problems

#### Topological gap-filling problem

A topological metabolic network comprised of *R* metabolic reactions and *M* metabolites is represented by a bipartite directed graph *G* = (*R* ∪ *M*, *E*) where edges in *E* connect either a metabolic reaction in *R* to metabolites in *M* or a metabolite in *M* to a metabolic reaction in *R*. Predecessors of a reaction node *r* are reactants: *reac*(*r*) = {*m* ∈ *M*|(*m*, *r*) ∈ *E*}. Successors of a reaction node are products: *prod*(*r*) = {*m* ∈ *M*|(*r*, *m*) ∈ *E*}. The biological concept of the producibility of metabolites can be formalized in terms of reachability. Given a metabolic network (*R* ∪ *M*, *E*) and a set *S* ⊆ *M* of seed metabolites, a reaction *r* ∈ *R* is reachable from the seeds *S* if all reactants in *reac*(*r*) are reachable from *S*. Pushing forward this definition, a metabolite *m* ∈ *M* is reachable from the set of seeds *S* if *m* ∈ *S* or if *m* ∈ *prod*(*r*) is the product of a reaction *r* ∈ *R* that is itself reachable from *S*. Following [25–27], the (forward) scope of a set of metabolites *S* according to a reaction set *R* contains all metabolites which can be iteratively produced from the seed metabolites. It is the set of metabolites reachable from *S* through the reactions in *R* and can be defined by iterating the set relation *M*_*i*+1_ = *M*_*i*_ ∪ *prod*({*r* ∈ *R*|*m* ∈ *reac*(*M*_*i*_), ∀*m*, (*r*, *m*) ∈ *E*}), with *M*_*O*_ = *S* until it reaches a fixed point. The scope can be computed in polynomial time.

We assume that we are given (i) a draft network *R*_*draft*_; (ii) a set *M*_*target*_ ⊂ *M* of target metabolites that are produced by the organism of interest; (iii) a set *M*_*seed*_ ⊂ *M* of seed nutrients found in the growth medium; and (iv) a database of metabolic reactions available to fill the network *R*_*database*_. The topological gap-filling problem is stated as follows.

$$\text{Minimize }Size(R_{fill}\;)\text{ s}.\text{t }\{\begin{array}{l} R_{fill}\subset R_{database}\\ M_{target}\cap scope_{R_{fill}\cup R_{draft}}(M_{seed})\text{ is maximal} \end{array}$$

This combinatorial problem, expressed as a logic encoding, is solved by the ASP solver clasp 3.0.5 [39] with domain specific heuristics *i.e.* search space exploration strategies, preserving correctness and completeness of the search procedure. Clasp allows the computation of the brave and cautious consequences (union and intersection of all solutions) by means of a linear number of calls to a solver (internally computing one solution) rather than enumerating the entirety of all solutions. This is accomplished by consecutive refinements of an internal constraint by relying on the incremental solving techniques introduced in [40]. Rather than computing a (possibly) exponentially increasing number of solutions, the idea is to compute a first solution, to record a constraint, eliminate it from further solutions, then to compute a second solution to strengthen the constraint in order to represent the intersection (or union) of the first two solutions, and to continue until no more solutions are obtained. This process involves computing at most as many solutions as there are logic variables in the input program. Depending on the chosen reasoning mode, either the cautious (properties shared by all solutions) or the brave (properties satisfied by at least a solution) consequences are then given by the variable assignment captured by the final constraint. This process enable all cardinal minimal solutions to be found but does not allow finding suboptimal solutions.

The ASP encoding, and the solver are encapsulated in a python package named Meneco [27, 41] which is freely available at http://bioasp.github.io/meneco. Experiments related to the *E. coli* benchmark were run on a node of 24 cores part of a computational cluster. Case studies were run on personal laptops: the computation time required to get the union of solutions was less than 30 seconds.

#### Stoichiometric gap-filling problem

A stoichiometric metabolic network can be viewed as an enrichment of a topological metabolic network with a stoichiometric matrix *S*. Under steady state conditions, metabolite balances yield ∑_*j*∈*R*_ *S*_*ij*_*v*_*j*_ = *b*_*j*_ for all *i* ∈ *M* where *b*_*i*_ is a parameter standing for uptake or secretion. In this setting, the stoichiometric gap-filling problem consists in minimizing the number of added reactions from the database to restore fluxes through a metabolite i* which is not producible from the draft metabolic network.

$$\text{Minimize }Size(R_{fill})\text{ s}.\text{t }.\;\{\begin{array}{l} \sum_{j\in R_{fill}\cup R_{draft}}S_{ij}v_{j}\leq 0\;\forall i\in M_{seed}\\ \sum_{j\in R_{fill}\cup R_{draft}}S_{ij}v_{j}=0\;\forall i\neq i*\\ \sum_{j\in R_{fill}\cup R_{draft}}S_{i*j}v_{j}>0 \end{array}$$

This problem is solved by the so-called GapFill tool, encoded in a GAMS (General Algebraic Modeling System) program [8].

To solve this optimization problem, solutions to the stoichiometric gap-filling problems for each individual target were computed by running the GAMS encoding from [8] on the NEOS server [42]. For each execution of the GAMS encoding, the program computed at most 30 sets of solutions to the gapfilling problem. The solution to the gap-filling problem was defined as the union of all reaction sets.

#### Subset-minimal gap-filling

The MIRAGE algorithm was run to perform a subset-minimal gap-filling using the implementation provided in [14]. Without any *a priori* information on the importance of the reactions in the draft networks, the biomass reaction was the only one added in the core reactions. The weights for all reactions of the databases were assigned to 1.

To be able to run the software on a computer cluster, the code was compiled with the mcc function of MATLAB (R2014a). The MATLAB runtime software (version 8.3) was used to run the compiled code on a computational cluster consisted of 138 computing nodes (1,700 cores).

#### Gap-filling and unblocking the entire models

The Portable System for the Analysis of Metabolic Models (PSAMM) [43] was used to run the fastGapFill algorithm. Cplex (version 12.6.2) was used as MILP solver to solve the optimisation problem. A weight of 100 was set for the addition of transport and import/export reactions to penalize any modifications of the models. The draft models were used as core sets.

### Networks and datasets

#### *E. coli* degraded metabolic networks

We analyzed the following GEMs of *E. coli*: *iJR*904 [44], *iAF*1260 [32] and *iJO*1366 [33]. The corresponding sbml files were downloaded from the associated publications or from the USCD repository USCD repository. To test the impact of the MetaCyc database on the gapfilling problem, cross-references between the SBML identifiers of metabolites (SBML species) in the networks and MetaCyc identifiers were created. To that end, we followed an iterative strategy based first on metabolite names and formulas, then on similar reactions.

For the three *E. coli* models *iJR*904, *iAF*1260 and *iJO*1366, we generated 90 random biomass functions obtained by setting a defined percentage of non-zero coefficients of the true biomass to zero. The choice of replaced non-zero coefficients relied on Bernoulli-distributed random binary numbers. For each model consisting of an *E. coli* metabolic network and a random biomass, we classified the metabolic reactions according to their functionality: blocked (flux values are always equal to 0 through the reaction), alternative (flux values are positive or equal to 0 through the reaction) and essential (flux values are always positive through the reaction). We implemented the classification of metabolic reactions within TOMLAB environment [45], using Gurobi as the underlying linear programming solver.

Then, for the three *E. coli* models *iJR*904, *iAF*1260 and *iJO*1366, we randomly removed 10%, 20%, 30% or 40% of metabolic reactions, with 10 different replicates by percentage. The same percentage of the three types of reactions was removed in each degradation process. The degraded networks were chosen such that none of the 90 random biomass functions could be produced by any of the 40 degraded networks.

All together, we obtained a set of 10,800 metabolic networks which were not functional with respect to the production of their associated biomass.

#### *E. siliculosus* draft metabolic network

EctoGEM 1.0 is based on genome data downloaded from the ORCAE website (http://bioinformatics.psb.ugent.be/orcae/overview/Ectsi) [46] on June 21, 2013; the *Ca.* P. ectocarpi genome used for network reconstruction is available from the European Nucleotide Archive (accession HG966617). Both networks were mapped against MetaCyc 19.0 as a reference database. The construction of the set of targets for Meneco relied on the dataset of 90,637 ESTs published in the *Ectocarpus* genome paper ([34], EMBL accessions: FP245546-FP312611).

#### *E. mutabilis* draft metabolic network

Sequences of *E. mutabilis* transcripts were obtained from *de novo* transcriptomic experiments published in [18]. The sequence data are publicly available in the Sequence Read Archive with the SRA accession numbers ERS358279 and ERS358280. The transcripts assembled with newbler were annotated by tblastn searches using reference enzyme sequences in MetaCyc 17.5 as queries. A reaction was included in the network if at least one of the corresponding reference enzyme sequences had a blast hit in the transcript set with an expect value lower than 10^−10^ and at least 25% identity over 80% of the length of the reference sequence.

Generic reactions in MetaCyc 17.5 involving generic carbohydrates, amino-acids, nucleotides or NAD-P-OR-NOP (standing for NAD or NADP) were replaced with the appropriately balanced instances involving the corresponding metabolites present in the *E. mutabilis* draft network. Transport and ambiguous non-informative reactions such as 3.4.14.10-RXN (POLYPEPTIDE + WATER → Peptides + TRIPEPTIDE) were discarded. The Meneco completion of the resulting draft metabolic network was performed with the modified version of MetaCyc 17.5 as the repair database and the 71 target metabolites listed in S3 Files. The version 17.5 of MetaCyc was used since it was the version available at the time this work was done, including manual addition of instances of generic reactions. Even though new versions of MetaCyc had been released during network reconstruction, we proceeded with version 17.5 for the sake of consistency.

#### *E. mutabilis* completion workflow

In order to avoid mutual dependencies and to solve cycles, the 72 targets were finally arranged into subsets corresponding to subnetworks (pathways and super-pathways) of the whole draft network. Completion was then performed with each subset as the list of targets for Meneco. At each step, seed metabolites included the basic mineral compounds and the metabolites required to unblock the cycles. Some metabolites such as ATP were added provisionally in order to solve long range dependencies until more information could be gathered about which reactions should be added to the network to enable their production. The production of the target subsets was addressed in the following order: photosynthesis, TCA cycle and amino-acids, glycolysis and glycogenesis, polyamines, nucleotides, and then all the remaining targets. Respiration was added as a single reaction.

When a reference enzyme sequence corresponding to an added reaction was available in the MetaCyc database, this sequence was used as a query to identify potential homologs in the *E. mutabilis* transcriptome by tblastn. Transcripts with an expect value lower than 10^−3^ were extracted and used to search the protein nr database with blastx on the NCBI web server. The presence of the reaction was validated if the search yielded significant hits (e-value < 10^−3^) in the Conserved Domain Database (CDD) and with sequences of enzymes that were consistent with the tested reaction.

After each step, if the added reactions produced new metabolites previously absent from the network, the spontaneous reactions described in MetaCyc consuming those metabolites were also added to the network. The appropriate instances were manually added for the generic reactions corresponding classes of the newly added metabolites.

### Functional studies

#### *E. coli* degraded networks after their completion

The Flux Balance Analyses studies were performed using the python-based toolbox COBRApy (version 0.3.2) [47] and the GLPK solver.

Flux Variability Analysis was performed using both COBRApy in association with GLPK, and homemade scripts within the TOMLAB environment using the Gurobi solver.

#### *E. mutabilis* metabolic network

The biomass reaction was defined according to [48] and based on the composition of *E. gracilis* as determined by [49–55]. The fatty acid composition of the *E. mutabilis* membrane adapted to arsenic contamination was obtained from [36]. The proportions of amino-acids and nucleotides in proteins and nucleic acids was estimated from the composition of transcripts and their translation.

After completion with Meneco, the network was tested with COBRA findBlockedReactions() command in Matlab R2015b. The dead-end metabolites blocking the production of biomass or of the experimentally determined targets [19] were manually identified and their boundaryCondition property was set to true. As transcripts corresponding to reactions that could regenerate reduced flavodoxins, oxidized ferredoxins, and thioredoxin, could be identified, the corresponding reactions (FLAVONADPREDUCT-RXN, RXN0-882, RXN-9944) were added to the network.

The resulting network was eventually tested in Flux Balance Analysis with COBRA in Matlab R2015b, using the BIOMASS-RXN reaction as the objective function, and the mineral seed metabolites listed in S3 Files as entries. Culture in the dark was simulated by setting the boundaries of the |Light| entry reaction (RXN_in_Light) to 0.

## Supporting Information

S1 Table Main characteristics of a panel of gap-filling methods. (PDF)

S1 Appendix Description of the different methods used in the benchmark and a more precised description of the obtained results. (PDF)

S1 Files *E. coli* benchmark of 10,800 non-functional degraded networks and FVA results. All files needed to run gap-filling experiments on the benchmark are provided in an archive as well as a Readme.txt file detailing the commands used to produce the results shown in this study. This archive also contains the global results for FVA and a detailed analysis of the network 256 and the biomass 89 as an example.
(ZIP)

S2 Files *E. siliculosus* networks and essential reactions. The two draft networks used to study potential cross-feeding relations between *E. siliculosus* and *Ca.* P. ectocarpi are provided together with the list of seeds and targets used to run the Meneco tool. The exhaustive analysis of essential reactions which allow the production of 83 target metabolites thanks to the combination of *E. siliculosus* and *Ca.* P. ectocarpi networks is provided in a separate file. Finally, detailled examples of false positive predicted interactions and their explanation are provided in a separate pdf file.
(ZIP)

S3 Files *E. mutabilis* networks. Both draft and functional reconstructed metabolic networks for *E. mutabilis* are provided here. The folder also provides the SBML file describing the repair database (MetaCyc 17.5 modified as described in the Material and Methods) required to run the Meneco tool. Files corresponding to targets and seeds are also provided. The file seed_minimum_medium_Emutabilis.sbml describes light and the 8 mineral nutrients of the minimal growth medium for *E. mutabilis*. As described in the main text, the network is not functional with only these seeds as reactions in cycles are blocked. The file “seeds_min_Emutabilis_final.sbml” provides the minimal set of seeds necessary to unblock the cycles and produce all the targets in “targets_Emutabilis_72.sbml” while the list of seeds and targets is also provided as tables in the file “seedsAndTargets_euglena.pdf”.
(ZIP)

## References

[1] Thiele I, Palsson BO. A protocol for generating a high-quality genome-scale metabolic reconstruction. Nature Protocols. 2010 1;5(1):93–121. Available from: http://www.pubmedcentral.nih.gov/articlerender.fcgi?artid=3125167&tool=pmcentrez&rendertype=abstract. 10.1038/nprot.2009.203 20057383 PMC3125167

[2] Henry CS, DeJongh M, Best AA, Frybarger PM, Linsay B, Stevens RL. High-throughput generation, optimization and analysis of genome-scale metabolic models. Nature Biotechnology. 2010 8;28(9):977–982. Available from: http://www.nature.com/doifinder/10.1038/nbt.1672. 10.1038/nbt.1672 20802497

[3] Agren R, Liu L, Shoaie S, Vongsangnak W, Nookaew I, Nielsen J. The RAVEN Toolbox and Its Use for Generating a Genome-scale Metabolic Model for *Penicillium chrysogenum*. PLoS Comput Biol. 2013 03;9(3):e1002980 Available from: http://dx.doi.org/10.1371%2Fjournal.pcbi.1002980. 23555215 10.1371/journal.pcbi.1002980 PMC3605104

[4] Vallenet D, Belda E, Calteau A, Cruveiller S, Engelen S, Lajus A, et al MicroScope—an integrated microbial resource for the curation and comparative analysis of genomic and metabolic data. Nucleic Acids Research. 2013;41(D1):D636–D647. Available from: http://nar.oxfordjournals.org/content/41/D1/D636.abstract. 10.1093/nar/gks1194 23193269 PMC3531135

[5] Karp PD, Paley S, Romero P. The Pathway Tools software. Bioinformatics. 2002 7;18(Suppl 1):S225–S232. Available from: http://bioinformatics.oxfordjournals.org/cgi/doi/10.1093/bioinformatics/18.suppl_1.S225. 10.1093/bioinformatics/18.suppl_1.S225 12169551

[6] Loira N, Zhukova A, Sherman DJ. Pantograph: A template-based method for genome-scale metabolic model reconstruction. Journal of Bioinformatics and Computational Biology. 2015;13(02):1550006 Available from: http://www.worldscientific.com/doi/abs/10.1142/S0219720015500067. 10.1142/S0219720015500067 25572717

[7] Handorf T, Ebenhöh O, Heinrich R. Expanding Metabolic Networks: Scopes of Compounds, Robustness, and Evolution. Journal of Molecular Evolution. 2005;61(4):498–512. Available from: 10.1007/s00239-005-0027-1. 16155745

[8] Satish Kumar V, Dasika MS, Maranas CD. Optimization based automated curation of metabolic reconstructions. BMC Bioinformatics. 2007;8:212 Available from: http://www.biomedcentral.com/1471-2105/8/212. 10.1186/1471-2105-8-212 17584497 PMC1933441

[9] Thiele I, Vlassis N, Fleming RMT. fastGapFill: efficient gap filling in metabolic networks. Bioinformatics (Oxford, England). 2014 9;30(17):2529–2531. Available from: 10.1093/bioinformatics/btu321. 24812336 PMC4147887

[10] Benedict MN, Mundy MB, Henry CS, Chia N, Price ND. Likelihood-Based Gene Annotations for Gap Filling and Quality Assessment in Genome-Scale Metabolic Models. PLoS Comput Biol. 2014 10;10(10):e1003882 Available from: http://dx.doi.org/10.1371%2Fjournal.pcbi.1003882. 25329157 10.1371/journal.pcbi.1003882 PMC4199484

[11] Mintz-Oron S, Meir S, Malitsky S, Ruppin E, Aharoni A, Shlomi T. Reconstruction of Arabidopsis metabolic network models accounting for subcellular compartmentalization and tissue-specificity. Proceedings of the National Academy of Sciences. 2012;109(1):339–344. Available from: http://www.pnas.org/content/109/1/339.abstract. 10.1073/pnas.1100358109 22184215 PMC3252957

[12] Herrgård MJ, Fong SS, Palsson BØ. Identification of Genome-Scale Metabolic Network Models Using Experimentally Measured Flux Profiles. PLoS Comput Biol. 2006 07;2(7):e72 Available from: http://dx.plos.org/10.1371%2Fjournal.pcbi.0020072. 10.1371/journal.pcbi.0020072 16839195 PMC1487183

[13] Kumar VS, Maranas CD. GrowMatch: An Automated Method for Reconciling In Silico In Vivo Growth Predictions. PLoS Comput Biol. 2009 03;5(3):e1000308 Available from: 10.1371/journal.pcbi.1000308. 19282964 PMC2645679

[14] Vitkin E, Shlomi T. MIRAGE: a functional genomics-based approach for metabolic network model reconstruction and its application to cyanobacteria networks. Genome biology. 2012;13(11):R111 Available from: 10.1186/gb-2012-13-11-r111. 23194418 PMC4053740

[15] Christian N, May P, Kempa S, Handorf T, Ebenhöh O. An integrative approach towards completing genome-scale metabolic networks. Molecular BioSystems. 2009 12;5(12):1889–903. Available from: http://pubs.rsc.org/en/content/articlehtml/2009/mb/b915913b. 10.1039/B915913b 19763335

[16] Monk J, Nogales J, Palsson BO. Optimizing genome-scale network reconstructions. Nature biotechnology. 2014;32(5):447–452. Available from: 10.1038/nbt.2870. 24811519

[17] Caspi R, Altman T, Billington R, Dreher K, Foerster H, Fulcher CA, et al The MetaCyc database of metabolic pathways and enzymes and the BioCyc collection of Pathway/Genome Databases. Nucleic Acids Research. 2014;42(D1):D459–D471. Available from: http://nar.oxfordjournals.org/content/42/D1/D459.abstract. 10.1093/nar/gkt1103 24225315 PMC3964957

[18] Halter D, Andres J, Plewniak F, Poulain J, Da Silva C, Arsène-Ploetze F, et al Arsenic hypertolerance in the protist Euglena mutabilis is mediated by specific transporters and functional integrity maintenance mechanisms. Environ Microbiol. 2015 6;17(6):1941–1949. Available from: 10.1111/1462-2920.12474. 24698441

[19] Halter D, Goulhen-Chollet F, Gallien S, Casiot C, Hamelin J, Gilard F, et al In situ proteo-metabolomics reveals metabolite secretion by the acid mine drainage bio-indicator, Euglena mutabilis. ISME J. 2012 7;6(7):1391–1402. Available from: 10.1038/ismej.2011.198. 22237547 PMC3379634

[20] Dittami SM, Barbeyron T, Boyen C, Cambefort J, Collet G, Delage L, et al Genome and metabolic network of “Candidatus Phaeomarinobacter ectocarpi” Ec32, a new candidate genus of Alphaproteobacteria frequently associated with brown algae. Frontiers in Genetics. 2014;5(241). Available from: http://www.frontiersin.org/systems_biology/10.3389/fgene.2014.00241/abstract. 10.3389/fgene.2014.00241 25120558 PMC4110880

[21] Prigent S, Collet G, Dittami SM, Delage L, Ethis de Corny F, Dameron O, et al The genome-scale metabolic network of Ectocarpus siliculosus (EctoGEM): a resource to study brown algal physiology and beyond. The Plant Journal. 2014;80(2):367–381. Available from: 10.1111/tpj.12627. 25065645

[22] Dittami SM, Eveillard D, Tonon T. A metabolic approach to study algal–bacterial interactions in changing environments. Molecular Ecology. 2014;23(7):1656–1660. Available from: 10.1111/mec.12670. 24447216

[23] Reed JL, Patel TR, Chen KH, Joyce AR, Applebee MK, Herring CD, et al Systems approach to refining genome annotation. Proc Natl Acad Sci U S A. 2006 11;103(46):17480–17484. Available from: 10.1073/pnas.0603364103. 17088549 PMC1859954

[24] Pharkya P, Burgard AP, Maranas CD. OptStrain: A computational framework for redesign of microbial production systems. Genome Research. 2004;14(11):2367–2376. Available from: http://genome.cshlp.org/content/14/11/2367.abstract. 10.1101/gr.2872004 15520298 PMC525696

[25] Romero PR, Karp P. Nutrient-related analysis of pathway/genome databases. Pac Symp Biocomput. 2001;p. 471–482. Available from: http://psb.stanford.edu/psb-online/proceedings/psb01/romero.pdf. 10.1142/9789814447362_0046 11262965

[26] Cottret L, Vieira Milreu P, Acuña V, Marchetti-Spaccamela A, Viduani Martinez F, Sagot MF, et al Enumerating Precursor Sets of Target Metabolites in a Metabolic Network In: Crandall K, Lagergren J, editors. Algorithms in Bioinformatics. vol. 5251 of Lecture Notes in Computer Science. Springer Berlin Heidelberg; 2008 p. 233–244. Available from: 10.1007/978-3-540-87361-7_20.

[27] Schaub T, Thiele S. Metabolic Network Expansion with Answer Set Programming In: International Conference on Logic Programming/Joint International Conference and Symposium on Logic Programming. Springer Berlin Heidelberg; 2009 p. 312–326. Available from: 10.1007/978-3-642-02846-5_27.

[28] Orth JD, Thiele I, Palsson BO. What is flux balance analysis? Nat Biotech. 2010 3;28(3):245–248. Available from: 10.1038/nbt.1614. PMC3108565 20212490

[29] Gebser M, Kaminski R, Kaufmann B, Schaub T. Answer set solving in practice. Synthesis Lectures on Artificial Intelligence and Machine Learning. 2012;6(3):1–238. Available from: 10.2200/S00457ED1V01Y201211AIM019.

[30] Marashi SA, Tefagh M. A mathematical approach to emergent properties of metabolic networks: partial coupling relations, hyperarcs and flux ratios. Journal of theoretical biology. 2014 8;355:185–193. Available from: 10.1016/j.jtbi.2014.04.011. 24751930

[31] de Figueiredo LF, Schuster S, Kaleta C, Fell DA. Can sugars be produced from fatty acids? A test case for pathway analysis tools. Bioinformatics. 2009;25(1):152–158. Available from: http://bioinformatics.oxfordjournals.org/content/25/1/152.abstract. 10.1093/bioinformatics/btn621 19117076

[32] Feist AM, Henry CS, Reed JL, Krummenacker M, Joyce AR, Karp PD, et al A genome-scale metabolic reconstruction for Escherichia coli K-12 MG1655 that accounts for 1260 ORFs and thermodynamic information. Mol Syst Biol. 2007 6;3 Available from: http://www.ncbi.nlm.nih.gov/pmc/articles/PMC1911197/. 10.1038/msb4100155 17593909 PMC1911197

[33] Orth JD, Conrad TM, Na J, Lerman JA, Nam H, Feist AM, et al A comprehensive genome-scale reconstruction of Escherichia coli metabolism–2011. Mol Syst Biol. 2011 10;7:535 Available from: http://www.ncbi.nlm.nih.gov/pmc/articles/PMC3261703/. 10.1038/msb.2011.65 21988831 PMC3261703

[34] Cock JM, Coelho SM, Brownlee C, Taylor AR. The Ectocarpus genome sequence: insights into brown algal biology and the evolutionary diversity of the eukaryotes. New Phytologist. 2010;188(1):1–4. Available from: 10.1111/j.1469-8137.2010.03454.x. 20840144

[35] Ye N, Zhang X, Miao M, Fan X, Zheng Y, Xu D, et al Saccharina genomes provide novel insight into kelp biology. Nature communications. 2015;6:6986 Available from: http://europepmc.org/articles/PMC4421812. 10.1038/ncomms7986 25908475 PMC4421812

[36] Halter D, Casiot C, Heipieper HJ, Plewniak F, Marchal M, Simon S, et al Surface properties and intracellular speciation revealed an original adaptive mechanism to arsenic in the acid mine drainage bio-indicator Euglena mutabilis. Applied Microbiology and Biotechnology. 2012;93(4):1735–1744. Available from: 10.1007/s00253-011-3493-y. 21792588

[37] Cottret L, Milreu PV, Acuña V, Marchetti-Spaccamela A, Stougie L, Charles H, et al Graph-Based Analysis of the Metabolic Exchanges between Two Co-Resident Intracellular Symbionts, Baumannia cicadellinicola and Sulcia muelleri, with Their Insect Host, Homalodisca coagulata. PLOS Computational Biology. 2010 09;6(9):1–13. Available from: http://dx.doi.org/10.1371%2Fjournal.pcbi.1000904. 20838465 10.1371/journal.pcbi.1000904 PMC2936742

[38] Loira N, Zhukova A, Sherman DJ. Pantograph: A template-based method for genome-scale metabolic model reconstruction. Journal of Bioinformatics and Computational Biology. 2015;13(02):1550006 Available from: http://www.worldscientific.com/doi/abs/10.1142/S0219720015500067. 25572717 10.1142/S0219720015500067

[39] Gebser M, Kaufmann B, Schaub T. Conflict-driven answer set solving: From theory to practice. Artificial Intelligence. 2012;187:52–89. Available from: http://www.sciencedirect.com/science/article/pii/S0004370212000409. 10.1016/j.artint.2012.04.001

[40] Gebser M, Kaminski R, Kaufmann B, Ostrowski M, Schaub T, Thiele S. Engineering an Incremental ASP Solver In: de la Banda MG, Pontelli E, editors. Proceedings of the Twenty-fourth International Conference on Logic Programming (ICLP’08). vol. 5366 of Lecture Notes in Computer Science. Springer; 2008 p. 190–205. Available from: 10.1007/978-3-540-89982-2_23.

[41] Collet G, Eveillard D, Gebser M, Prigent S, Schaub T, Siegel A, et al Extending the Metabolic Network of *Ectocarpus Siliculosus* Using Answer Set Programming In: Cabalar P, Son TC, editors. Proceedings of the Twelfth International Conference on Logic Programming and Nonmonotonic Reasoning. vol. 8148 of Lecture Notes in Computer Science. Springer; 2013 p. 245–256. Available from: 10.1007/978-3-642-40564-8_25.

[42] Czyzyk J, Mesnier MP, More JJ. The NEOS Server. Computational Science Engineering, IEEE. 1998 7;5(3):68–75. Available from: 10.1109/99.714603.

[43] Steffensen JL, Dufault-Thompson K, Zhang Y. PSAMM: A Portable System for the Analysis of Metabolic Models. PLoS Comput Biol. 2016 02;12(2):1–29. Available from: http://dx.doi.org/10.1371%2Fjournal.pcbi.1004732. 26828591 10.1371/journal.pcbi.1004732 PMC4734835

[44] Reed JL, Vo TD, Schilling CH, Palsson BO. An expanded genome-scale model of Escherichia coli K-12 (iJR904 GSM/GPR). Genome Biol. 2003;4(9):R54 Available from: http://www.ncbi.nlm.nih.gov/pmc/articles/PMC193654/. 10.1186/gb-2003-4-9-r54 12952533 PMC193654

[45] Kenneth H. The TOMLAB Optimization Environment in Matlab. Advanced Modeling and Optimization. 1999;1(1):47–69. Available from: http://tomopt.com/docs/Tomlab-v1.0-Advanced-Modeling-and-Optimization-1999-vol-1-num-1.pdf.

[46] Sterck L, Billiau K, Abeel T, Rouzé P, Van de Peer Y. ORCAE: online resource for community annotation of eukaryotes. Nature methods. 2012 11;9(11):1041 Available from: 10.1038/nmeth.2242. 23132114

[47] Ebrahim A, Lerman J, Palsson B, Hyduke D. COBRApy: COnstraints-Based Reconstruction and Analysis for Python. BMC Systems Biology. 2013;7(1):74 Available from: http://www.biomedcentral.com/1752-0509/7/74. 23927696 10.1186/1752-0509-7-74 PMC3751080

[48] Förster J, Famili I, Fu P, Palsson BO, Nielsen J. Genome-Scale Reconstruction of the Saccharomyces cerevisiae Metabolic Network. Genome Research. 2003;13(2):244–253. Available from: http://genome.cshlp.org/content/13/2/244.abstract. 10.1101/gr.234503 12566402 PMC420374

[49] Buetow D, Levedahl B. Decline in the cellular content of RNA, protein and dry during the logarithmic growth of Euglena gracilis. Microbiology. 1962;28(4):579–584. Available from: http://mic.microbiologyresearch.org/content/journal/micro/10.1099/00221287-28-4-579. 13874466 10.1099/00221287-28-4-579

[50] Kott Y, Wachs AM. Amino acid composition of bulk protein of Euglena grown un water. Applied Microbiology. 1964 7;12(4):292–294. Available from: http://www.ncbi.nlm.nih.gov/pmc/articles/PMC1058119/. 14199015 10.1128/am.12.4.292-294.1964 PMC1058119

[51] Constantopoulos G, Bloch K. Effect of Light Intensity on the Lipid Composition of Euglena gracilis. Journal of Biological Chemistry. 1967;242(15):3538–3542. Available from: http://www.jbc.org/content/242/15/3538.abstract.

[52] Constantopoulos G. Lipid metabolism of manganese-deficient algae. I. Effect of manganese deficiency on the greening and the lipid composition of Euglena gracilis Z. Plant Physiology. 1970 1;45(1):76–80. Available from: http://www.ncbi.nlm.nih.gov/pmc/articles/PMC396358/. 10.1104/pp.45.1.76 5436328 PMC396358

[53] Terry OW, Edmunds LN. Phasing of cell division by temperature cycles in Euglena cultured autotrophically under continuous illumination. Planta. 1970;93(2):106–127. Available from: 10.1007/BF00387119. 24496707

[54] Regnault A, Chervin D, Chammai A, Piton F, Calvayrac R, Mazliak P. Lipid composition of Euglena gracilis in relation to carbon-nitrogen balance. Phytochemistry. 1995;40(3):725–733. Available from: http://www.sciencedirect.com/science/article/pii/003194229500268C. 10.1016/0031-9422(95)00268-C

[55] Lunau M, Lemke A, Walther K, Martens-Habbena W, Simon M. An improved for counting bacteria from sediments and turbid environments by epifluorescence microscopy. Environmental Microbiology. 2005;7(7):961–968. Available from: 10.1111/j.1462-2920.2005.00767.x. 15946292

