# Supplementary material for: Meneco, a Topology-Based Gap-Filling Tool Applicable to Degraded Genome-Wide Metabolic Networks
Source: PLoS Comput Biol. 2017 Jan 27;13(1):e1005276. doi: 10.1371/journal.pcbi.1005276 (PMC5302834; doi:10.1371/journal.pcbi.1005276)
Supplement: S1 Table — (PDF) [file pcbi.1005276.s001.pdf]

Table 1. Main characteristics of a panel of gap-filling methods

Name	Input data	Technological platform	Application models
**GapFill [1]**	Target metabolites	GAMS platform	*Escherichia coli* strains,<br>*Gordonia alkanivorans* [2]<br>*Salmonella Typhimurium LT2* [3]<br>*Clostridium beijerinckii NCIMB 8052* [4]
**FastGapFill [5]**	Target metabolites	Matlab & Cplex	*E. coli* [5]<br>*Synechocystis sp.* [5]
**Meneco**	Target metabolites	Python	*Ectocarpus siliculosus* [6]<br>*Candidatus* Phaeomarinobacter ectocarpi [7]
**Christian *et al.* [8]**	Target metabolites<br>Likelihood of reactions	No available distribution	*E. coli* [8]<br>*Ostreococcus* [9]<br>*Chlamydomonas reinhardtii* [8]
**Benedict *et al.* [10]**	Target metabolites<br>Likelihood of reactions	KBase framework	21 bacterial species [10]
**Mintz-Oron *et al.* [11]**	Target metabolites<br>Proteomic data	No available distribution	*Arabidopsis thaliana* [11]
**Mirage [12]**	Target metabolites<br>Taxonomic weights<br>Co-expression transcriptomic weights	Matlab	*Cyanobacteria* [12]
**Growmatch [13]**	Targeted metabolites<br>Growth/no growth data	Matlab	*Methanosarcina acetivorans* [14]<br>*Saccharomyces cerevisiae* [15]
**OMNI [16]**	Flux data	Matlab & GAMS	*E. coli* [16]

References

1. Satish Kumar V, Dasika MS, Maranas CD. Optimization based automated curation of metabolic reconstructions. BMC Bioinformatics. 2007;8:212. Available from: http://www.biomedcentral.com/1471-2105/8/212.

2. Aggarwal S, Karimi IA, Reinaldi Ivan G. In silico modeling and evaluation of Gordonia alkanivorans for biodesulfurization. Mol BioSyst. 2013;9:2530–2540. Available from: http://dx.doi.org/10.1039/C3MB70132H.

3. Thiele I, Hyduke DR, Steeb B, Fankam G, Allen DK, Bazzani S, et al. A community effort towards a knowledge-base and mathematical model of the human pathogen Salmonella Typhimurium LT2. BMC Systems Biology. 2011;5(1):1–9. Available from: http://dx.doi.org/10.1186/1752-0509-5-8.

4. Milne CB, Eddy JA, Raju R, Ardekani S, Kim PJ, Senger RS, et al. Metabolic network reconstruction and genome-scale model of butanol-producing strain Clostridium beijerinckii NCIMB 8052. BMC Systems Biology. 2011;5(1):1–15. Available from: http://dx.doi.org/10.1186/1752-0509-5-130.

5. Thiele I, Vlassis N, Fleming RMT. fastGapFill: efficient gap filling in metabolic networks. Bioinformatics (Oxford, England). 2014 Sep;30(17):2529–2531. Available from: http://dx.doi.org/10.1093/bioinformatics/btu321.

6. Prigent S, Collet G, Dittami SM, Delage L, Ethis de Corny F, Dameron O, et al. The genome-scale metabolic network of Ectocarpus siliculosus (EctoGEM): a resource to study brown algal physiology and beyond. The Plant Journal. 2014;80(2):367–381. Available from: http://dx.doi.org/10.1111/tpj.12627.

7. Dittami SM, Barbeyron T, Boyen C, Cambefort J, Collet G, Delage L, et al. Genome and metabolic network of "Candidatus Phaeomarinobacter ectocarpi" Ec32, a new candidate genus of Alphaproteobacteria frequently associated with brown algae. Frontiers in Genetics. 2014;5(241). Available from: http://www.frontiersin.org/systems_biology/10.3389/fgene.2014.00241/abstract.

8. Christian N, May P, Kempa S, Handorf T, Ebenhöh O. An integrative approach towards completing genome-scale metabolic networks. Molecular BioSystems. 2009 Dec;5(12):1889–903. Available from: http://pubs.rsc.org/en/content/articlehtml/2009/mb/b915913b.

9. Krumholz EW, Yang H, Weisenhorn P, Henry CS, Libourel IGL. Genome-wide metabolic network reconstruction of the picoalga Ostreococcus. Journal of Experimental Botany. 2011;Available from: http://jxb.oxfordjournals.org/content/early/2011/12/28/jxb.err407.abstract.

10. Benedict MN, Mundy MB, Henry CS, Chia N, Price ND. Likelihood-Based Gene Annotations for Gap Filling and Quality Assessment in Genome-Scale Metabolic Models. PLoS Comput Biol. 2014 10;10(10):e1003882. Available from: http://dx.doi.org/10.1371%2Fjournal.pcbi.1003882.

11. Mintz-Oron S, Meir S, Malitsky S, Ruppin E, Aharoni A, Shlomi T. Reconstruction of Arabidopsis metabolic network models accounting for subcellular compartmentalization and tissue-specificity. Proceedings of the National Academy of Sciences. 2012;109(1):339–344. Available from: http://www.pnas.org/content/109/1/339.abstract.

12. Vitkin E, Shlomi T. MIRAGE: a functional genomics-based approach for metabolic network model reconstruction and its application to cyanobacteria networks. Genome biology. 2012;13(11):R111. Available from: http://dx.doi.org/10.1186/gb-2012-13-11-r111.

13. Kumar VS, Maranas CD. GrowMatch: An Automated Method for Reconciling In Silico In Vivo Growth Predictions. PLoS Comput Biol. 2009 03;5(3):e1000308. Available from: http://dx.doi.org/10.1371/journal.pcbi.1000308.

14. Satish Kumar V, Ferry JG, Maranas CD. Metabolic reconstruction of the archaeon methanogen Methanosarcina Acetivorans. BMC Systems Biology. 2011;5(1):1–10. Available from: http://dx.doi.org/10.1186/1752-0509-5-28.

15. Zomorrodi AR, Maranas CD. Improving the iMM904 S. cerevisiae metabolic model using essentiality and synthetic lethality data. BMC Systems Biology. 2010;4(1):1–15. Available from: http://dx.doi.org/10.1186/1752-0509-4-178.

16. Herrgård MJ, Fong SS, Palsson BØ. Identification of Genome-Scale Metabolic Network Models Using Experimentally Measured Flux Profiles. PLoS Comput Biol. 2006 07;2(7):e72. Available from: http://dx.plos.org/10.1371%2Fjournal.pcbi.0020072.
