# Supplementary material for: Meneco, a Topology-Based Gap-Filling Tool Applicable to Degraded Genome-Wide Metabolic Networks
Source: PLoS Comput Biol. 2017 Jan 27;13(1):e1005276. doi: 10.1371/journal.pcbi.1005276 (PMC5302834; doi:10.1371/journal.pcbi.1005276)
Supplement: S1 Appendix — (PDF) [file pcbi.1005276.s002.pdf]

Supplementary material

1 Characteristics of gap-filling methods and precise description of the benchmark

The table 1 presents the characteristics of different methods used in this benchmark to compare the completion of metabolic networks.

The four tools were applied to complete the 3,600 GEMs of our benchmark with the MetaCyc reference database, to check which methods were applicable in practice. The enumeration of all solutions to the parsimonious topological gap-filling problem with Meneco ended in 3,326 cases over the 3,600 studied cases. Our computation reported that there are 1,798 solution sets on average (minimum: 1, maximum: 829,440), suggesting that many combinations of pathways may restore the production of all targets considered together. Then we defined the output of Meneco to be the set of all reactions appearing in at least one of the minimal sets. Using an efficient solving strategy, the output could be computed in three minutes on average on a single core. The computational time of fastGapFill to report a single set of reactions to be added to each of the 3600 GEM was, on average, between one and two minutes. This computational time to obtain an unique solution was equivalent to those of Meneco (that gets the union of all possible solution) as soon an efficient MILP solver was used.

The number of enumerated solutions to the topological parsimonious problem confirmed that GapFill could not be used to perform an exhaustive gap-filling of the GEMs in practice. This is due to to complexity of the MILP problem solved that forced us to bound the number of solutions reported by GapFill by a too low parameter value to be significant. Because of this negative bias for GapFill, this tool was not included in the comparison of the methods using the MetaCyc database.

In terms of computational time for solving, we noticed that an important gap exists between MIRAGE and the two other tools for algorithmic reasons. More precisely, for a chosen GEM, the MIRAGE algorithm had to be run 100 times to rank the reactions. This ranking was used in a last run of the algorithm to report a final solution to the MIRAGE gap-filling problem. In average, one iteration of the algorithm per GEM lasted around 30 minutes when completing with MetaCyc. Since the algorithm has to be run at least 100 times, a computer cluster is required to use MIRAGE on a real world case. In order to obtain an estimate of the variability of solutions reported by the algorithm, we defined the output of MIRAGE for a GEM to be the union of each set of reactions reported by 100 different runs of the 101th iteration. Limited by computational performances, such outputs were computed for a sample of 360 GEMs of our benchmark. Results of the 100 runs were globally consistent over the MetaCyc database as 63% of the reactions of the union appeared in at least 75% of the solutions. Yet the sizes of the unions of solution were too large to be interpreted and manually curated, with an average of 4,029 reactions (minimum=3,481, maximum=4,228) for a metabolic network with an initial size of 1,075 reactions (Fig. 1). Note that the average size of one solution among the 100 ones proposed by the union is 2,976 (±1,151) reactions. This analysis suggests that MIRAGE is not suited to be used with minimal data (draft, seeds, targets and no a priori scoring on the database) for gap-filling and that the algorithm needs all the recommended data (phylogenetic and/or transcriptomic scores) to perform correctly

Table 1. Characteristics of gap-filling methods. Gap-filling methods mainly differ with respect to the set of modifications to the system they enable (approximating the criteria of producibility, changing the reversibility of reactions, modification of import/export reactions). Therefore, they can be classified according to (i) the set of compounds whose producibility should be restored; (ii) the criteria they optimize and (iii) the number of solutions set they return.

	Which aim?	Which problem is solved?	Which exploration of the search space?
GapFill	The stoichiometry-based production of a single target is enabled by adding a minimal number of reactions from the reference database.	The problem is modeled by a MILP optimization problem which forces the production of target fluxes, encoded in a GAMS program.	The algorithm reports a bounded (parameterized) number of solutions to the gapfilling problem, ordering them by the number of reactions they contain.
Meneco	The graph-based simultaneous production of a set of multiple targets is enabled by adding a minimal number of reactions from the reference database.	The problem is approximated by a combinatorial optimization problem describing topological constraints for the production of a metabolite and solved with Answer Set Programming technologies.	The algorithm reports an exhaustive enumeration of all solutions of minimal size for the complete set of targets. It also reports the global solution set consisting of all reactions appearing in at least one solution.
fast-GapFill	All reactions from the draft model are unblocked by selecting a minimal number of reactions from the reference database or import/export fluxes for internal metabolites.	The problem is solved by computing a near-minimal set of reactions that need to be added to the draft metabolic network to render it flux consistent (FastCore algorithm). The search is modeled by MILP optimization problems solved with Cplex.	The algorithm reports a single solution. The network, both enriched with additional reactions from the reference database and modified according to novel import fluxes, has no blocked reactions with respect to its core set.
MIRAGE	The flux of a set of multiple reactions (including biomass) is enabled by adding a set of reactions from the reference database. The algorithm favors reactions whose presence is supported by additional data, when available.	The top-down algorithm randomly identifies a set of reactions from the reference database in which all reactions have a non-zero flux. An iterative procedure selects reactions to be removed until the model is no more functional. A ranking of reactions according to their impact of flux distributions is obtained by applying the procedure a parameterized number of times.	The algorithm reports a single set of reactions which enables the flux production of all target fluxes. The model is no longer functional when removing any of the reported reaction (subset minimality). As the algorithm is not deterministic, the reported solution may change at each application of the algorithm.

and gain robustness, thus reducing the size of the output. However this kind of information is often sparse or nonexistent for non-usual species, to which Meneco aims to be applied.

Functional study of GEMs completed by fastGapFill, Meneco and MIRAGE. Due to the drawbacks of gap-filling methods, networks filled by both Meneco and fastGapFill may not be functional. Meneco does not take into account constraints of mass-balance equations, so that the compound T_1 of Fig. 1 (main manuscript) cannot be produced when the completion is based on the reaction R_9. In contrast, the MILP problem solved by fastGapFill introduces export and import reactions that may not be biologically relevant and must be removed from the output of the method. In [1], Latendresse *et al* altered GEMs by removing 1 to 14 reactions and checked whether their tool could recover these reactions and the global functionality of the network. Inspired by this method, in Fig. 1, we compared both the number of reactions contained in the output of Meneco, fastGapFill and MIRAGE for the tested networks and the number of completed GEMs which recovered the biomass synthesis capability using Flux Balance Analysis (FBA).

Fig 1. Comparison of the sizes of the output of the three gap-filling methods Meneco, fastGapFill, MIRAGE. From 360 (MIRAGE) to 3,600 degraded GEMs (fastGapFill, Meneco) were completed with the gap-filling algorithms using the MetaCyc reference database. GEMs were gathered according to their initial size (90%, 80%, 70% and 60% of the ***iJR*904** *E. coli* GEM). The number of reactions introduced in each GEM to restore its functionality is compared to the number of reactions removed from the original network and the capability of the completed GEM to restore the producibility of biomass (FBA).

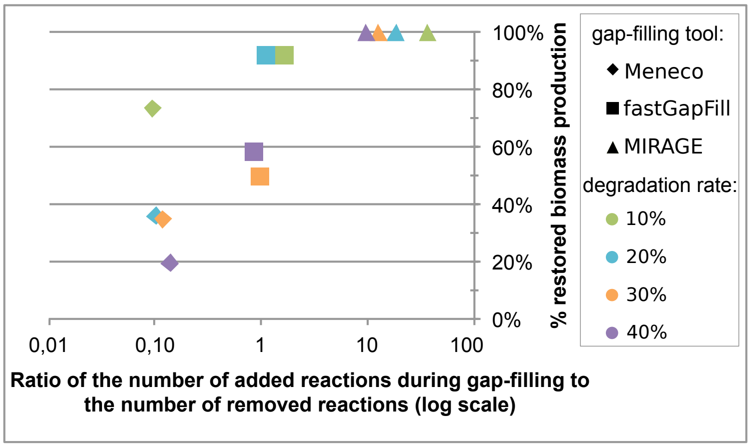

As expected given the large sizes of the proposed completions, 100% of GEMs filled by the union of MIRAGE results recovered the biomass synthesis. We noticed that the size of the MIRAGE output ranged from 3,481 to 4,228 reactions (average value: 4,028), although the initial reconstructed network contained 1,075 reactions. This result is out of range and suggests that MIRAGE cannot be used to complete a GEM without additional evidences for reactions.

For each GEM, the output of fastGapFill contained on average 87 import or export reactions (minimum = 72, maximum = 108) which were not contained in the reference database. However, in the case that all available metabolites are known (*i.e.* growth

medium), it might seem non-relevant to import other internal compounds to unblock fluxes. To be able to compare the fastGapFill results with the other methods we therefore removed import and export fluxes form the solutions. 72.88% of the so-completed GEMs by fastGapFill recovered their biomass synthesis ability, with very high rates for 10% and 20% degraded networks. Altogether, fastGapFill added 273 reactions in average (minimum = 150, maximum = 388). This is slightly more than the number of reactions initially removed from the network (+14% in average, +64% for 10% degraded networks) (Fig. 1). This suggests that fastGapFill is very efficient to restore the functionality of a network but that a large number of reactions proposed by fastGapFill should be manually curated before being usable.

A characteristic of Meneco is the very small size of its outputs, which contained from 0 to 110 reactions (32 on average), in line with the parsimonious criteria used. This is less than 15% of the number of reactions removed from the original networks (Fig. 1). Yet despite the very low number of added reactions, in average, 40.83% of the networks completed with Meneco recovered the capability of synthesizing biomass. Altogether, 73% of GEMs with a 10% degradation rate in our benchmark became functional after gap-filling. This suggests that the Meneco tool finds a reasonable trade-off between the size of the output to enable a manual curation and the biological significance for genomes and transcriptomes produced by NGS technologies.

The results of the completion procedure showed that the choice of the reference database has a low impact on the size of the Meneco output. Meneco returned solution sets of relatively small size (from 0 to 64 reactions for the $iJR904$ network, from 0 to 126 reactions for $iAF1260$, from 0 to 107 reactions for $iJO1366$). On average, Meneco added only 23 reactions (2.1% of original network size) to the $iJR904$ network, 41.5 reactions (1.7%) to the $iAF1260$ network, and 33.5 reactions (1.3%) to the $iJO1366$ network. The size of the solution set increased linearly with the degradation rate of the network (see Appendix S2 Section 1) and independently of the reference database.

For each completed GEM, we checked whether biomass production was restored in FBA. Our results suggest that the quality of the network has a strong impact on the performance of Meneco. For the $iJR904$ network, Meneco succeeded in 975 among the 3,600 cases. For the $iAF1260$ and the $iJO1366$ networks, Meneco managed to reconstruct a functional network in 1,198 and 1,315 cases respectively. In particular, the rates of success for 10% and 20% degraded GEMs highly depend on the chosen database (Fig. 4 (main text), see also Appendix S2 Section 1).

For the $iJR904$ highly degraded (40%) networks, we noticed that the gap-filling procedure succeeded only in 3,2% of cases. On the contrary, our previous experimentations depicted in Table 1 (main text) evidenced that, for the same degraded GEMs, biomass producibility was restored in 19% of the cases when using a reference database consisted of the union of MetaCyc database and the initial $iJR904$ (see also Appendix S2, Section 4). These results show that the performance and the accuracy of gap-filling methods depend on the content and quality of the reference database. This is a major drawback for the study of real-world problems where missing reactions may not be among well-known metabolic pathways. Based on this consideration, it should be acknowledged that the size of gap-filling completions cannot be considered as a measure of accuracy of the method.

2 Gap-filling 10,800 *E. coli* degraded GEMs with parsimonious tools Meneco and GapFill

Degradation and completion protocol In order to study the accuracy of topologic and stoichiometric-based parsimonious gap-filling methods, we created a

benchmark of 10,800 degraded GEMs from the three GEMs $iJR904$, $iAF1260$ and $iJO1366$. This benchmark was specifically used to test whether parsimonious gap-filling approaches could recover the functionalities of reactions in a well-understood network although they select much fewer reactions than the number of removed reactions.

We analyzed the following *E. coli* GEMs: $iJR904$, $iAF1260$ and $iJO1366$. For each of them, a benchmark consisting of three sets of 90 biomass functions combined with 40 networks (obtained by removing 10%, 20%, 30%, or 40% of the initial network) was built. Degradation occurred through all types of pathways, including the central ones, such as the Tri-Carboxylic Acid cycle (TCA) (See section 6). None of the 10,800 (40*90*3) degraded networks was capable of producing the corresponding biomass.

For each combination of degraded *E. coli* network and biomass composition, Meneco and GapFill were applied to complete the network in order to produce all compounds included in the biomass. Note that, in particular, GapFill was not used to produce all blocked metabolites present in the network, as it could have been done using the GapFind algorithm, but only those involved in the functional growth of the network.

For Meneco, seeds were defined to be external compounds of the initial *E. coli* models. The reference database contained all reactions from the initial *E. coli* models. To solve the topological gap-filling problem, we first enumerated all sets of reactions with a minimal size which allowed to simultaneously restore the topological producibility of all compounds in the associated biomass. Our computation reported that there are 1,798 solution sets on average (minimum: 1, maximum: 829,440), suggesting that many combinatorial combinaisons of pathways may restore the production of all targets considered together. Then we defined the output to the be the set of all reactions appearing in at least one of the minimal sets.

When applied to the considered networks and individual targets, GapFill identified all completion sets of minimal size together with several completion sets of minimal size +1. The parameter for the maximum number of completion sets was set to 30. This threshold was chosen according to preliminary work and appeared to be sensible since only 68 networks reached the limit of 30 completion sets among the 10,800 degraded networks. The union of all completion sets for each individual target was defined to be the solution of the multi-target gap-filling problem. This was motivated by the fact that GapFill requires an upper limit with respect to the number of reported solutions, which can explode up to 800,000 according to the Meneco analysis, when considering all targets at the same time. This choice was confirmed by additional experiments showing that adding an artificial reaction consuming all targets and defining the output of this reaction as the new single target had a significant negative impact on the quality of the results (only 64% of essential reactions of the $iAF1260$ network were recovered on average instead of 83%). By extension of the gap-filling problem, we assume that we are given a set of seed metabolites M_{seed} and a set of targeted metabolites M_{target}. The multi-target gap-filling problem consists in identifying sets of reactions that restore the production of all metabolites in M_{target} provided that the metabolites taken up belong to M_{seed}. The solution to the multi-target gap-filling problem was defined as the union of all reaction sets.

Size of completion datasets The results of these gap-filling procedures (Table 2 for the complete benchmark, Tables 3 to 6 for each degradation rates) show that although a large number of reactions was removed from the network, both Meneco and GapFill returned solution sets of relatively small size (from 0 to 64 reactions for the $iJR904$ network, from 0 to 126 reactions for $iAF1260$, from 0 to 107 reactions for $iJO1366$). On average, Meneco or GapFill added only 23 reactions (2.1%) to the $iJR904$ network, 41.5 reactions (1.7%) to the $iAF1260$ network, and 33.5 reactions (1.3%) to the

*iJO*1366 network. On average, Meneco returned 1.6% fewer reactions (*i.e.* 4) than GapFill. Interestingly, although Meneco and GapFill solutions are comparable in sizes, they only share 45.3% of the reactions in their contents (see section 3).

We analyzed the size of GapFill and Meneco completion with regards to the degradation rates of draft networks. There is a correlation between both: the size of the completion tends to grow when the degradation rate rises (Fig. 2).

Table 2. Characteristics of the 10,800 networks in the benchmark of *E. coli* degraded GEMs and their completion with Meneco and GapFill. Between 10% and 40% of reactions were removed from three *E. coli* reference networks in order to block the FBA-based production of 40 different biomass functions. For each degraded network, both the Meneco and the GapFill tools were used to restore the producibility of the biomass. The same table is available for the four different degradation rates in Section 1.

Reference network	***iJR*904**	***iAF*1260**	***iJO*1366**
**Characteristics**			
reactions	1075	2383	2582
compounds	1800	1967	2129
**Removed reactions**			
min	101	216	239
max	446	960	1055
mean	269	597	650
**Essential reactions removed**			
min	0	0	0
max	21	28	27
mean	5.18	5.81	4.58
**Blocked reactions removed**			
min	15	18	27
max	112	108	140
mean	55	57	75
**Alternative reactions removed**			
min	72	187	199
max	349	870	943
mean	208	533	569
**Reactions added by Meneco**			
min	0	4	0
max	64	90	90
mean	23	35	30
**Reactions added by GapFill**			
min	0	0	0
max	63	126	107
mean	23	48	37

Degradation and recovery of Tri-Carboxylic Acid cycle in gap-filling experiments To have better insights into Meneco and GapFill behaviours towards cycles, we studied the degradation of the Tri-Carboxylic Acid cycle (TCA cycle) in each of the 120 (40 networks for each of the three E. coli) degraded networks and its potential recovery in the 10,800 network completions. 105 networks out of 120 (88%) had a degraded TCA cycle, with the number of missing reactions ranging from 1 to 7 (Fig. 3). Neither Meneco nor GapFill succeeded in recovering all the missing TCA reactions for a network (data not shown). When only one reaction was missing, it was never recovered by any of the tools. There was a total of 26,820 missing TCA reactions distributed over 9,450 networks (out of 10,800). Meneco recovered 627 reactions and GapFill 1,474. Both results are insignificant regarding the total number of reactions (respectively 2.3% and 5.4%). When studying these results in parallel to the biomass production restoration, it turned out that there was no relationship between the two features. Parsimonious gap-filling is independent from central metabolism pathways completion to the extent that there exists shorter paths in the network to produce the targets.

Table 3. Characteristics of the 2,800 networks with 10% degradation rate.

Reference network	*iJR*904	*iAF*1260	*iJO*1366
**Characteristics**			
reactions	1075	2383	2582
compounds	1800	1967	2129
**Removed reactions**			
min	101	217	239
max	117	249	287
mean	109	237	260
**Essential reactions removed**			
min	0	0	0
max	7	11	8
mean	2.19	2.50	1.83
**Blocked reactions removed**			
min	15	18	27
max	28	30	38
mean	22	23	31.48
**Alternative reactions removed**			
min	72	187	199
max	92	227	256
mean	84	211	225
**Reactions added by Meneco**			
min	0	4	0
max	10	18	11
mean	6.99	9.09	6.43
**Reactions added by GapFill**			
min	0	0	0
max	11	18	12
mean	4.44	19.14	7.26

Table 4. Characteristics of the 2,800 networks with 20% degradation rate.

Reference network	*ijR*904	*iAF*1260	*iJO*1366
**Characteristics**			
reactions	1075	2383	2582
compounds	1800	1967	2129
**Removed reactions**			
min	203	513	502
max	226	471	530
mean	216	486	517
**Essential reactions removed**			
min	0	0	0
max	13	18	14
mean	4.67	4.73	3.33
**Blocked reactions removed**			
min	32	33	45
max	61	63	77
mean	44	47	59
**Alternative reactions removed**			
min	144	411	425
max	177	462	473
mean	166	434	453
**Reactions added by Meneco**			
min	3	10	6
max	24	40	33
mean	16	26	19
**Reactions added by GapFill**			
min	1	6	2
max	29	49	37
mean	13.5	32.59	24

Table 5. Characteristics of the 2,800 networks with 30% degradation rate.

Reference network	***iJR*904**	***iAF*1260**	***iJO*1366**
**Characteristics**			
reactions	1075	2383	2582
compounds	1800	1967	2129
**Removed reactions**			
min	307	696	776
max	343	738	808
mean	325	717	788
**Essential reactions removed**			
min	0	0	0
max	18	24	20
mean	6.47	7.38	5.56
**Blocked reactions removed**			
min	59	56	77
max	81	81	108
mean	66	69	90
**Alternative reactions removed**			
min	227	605	662
max	276	667	721
mean	252	640	692
**Reactions added by Meneco**			
min	3	24	20
max	40	70	68
mean	27.8	43.4	37.9
**Reactions added by GapFill**			
min	1	9	8
max	40	97	71
mean	21.2	62	47.63

Table 6. Characteristics of the 2,800 networks with 40% degradation rate.

Reference network	***iJR*904**	***iAF*1260**	***iJO*1366**
**Characteristics**			
reactions	1075	2383	2582
compounds	1800	1967	2129
**Removed reactions**			
min	414	932	999
max	446	961	1055
mean	430	948	1036
**Essential reactions removed**			
min	0	0	0
max	21	28	27
mean	7.83	8.65	7.62
**Blocked reactions removed**			
min	75	79	104
max	112	108	140
mean	89	91	120
**Alternative reactions removed**			
min	304	808	840
max	349	870	943
mean	332	848	906
**Reactions added by Meneco**			
min	12	41	36
max	64	90	90
mean	42.1	62.8	57.68
**Reactions added by GapFill**			
min	7	41	19
max	63	126	107
mean	38.1	88.5	68.52

Fig 2. Degradation rates and completion sizes for the *E. coli* benchmark
X-axis depicts the degradation rate for each of the three networks and Y-axis the sizes of completions for Meneco and GapFill.

(a) $iJR904$

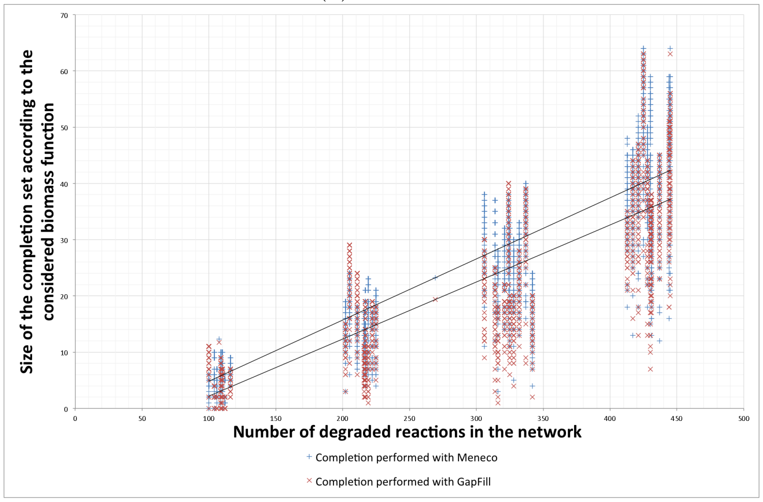

(b) $iAF1260$

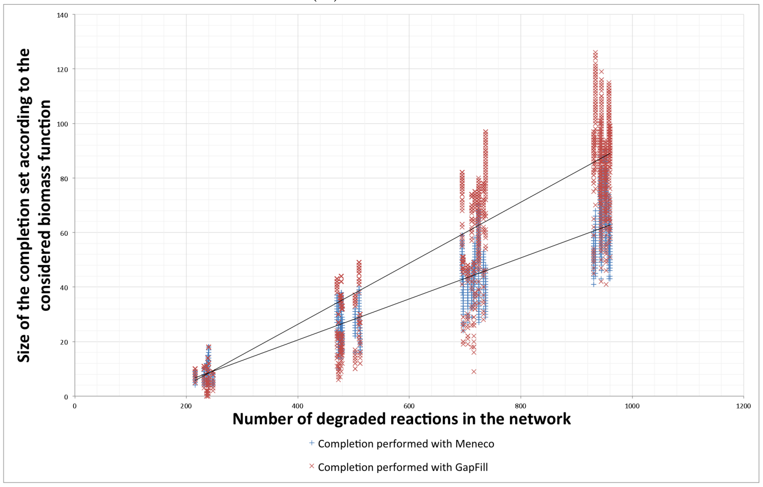

(c) $iJO1366$

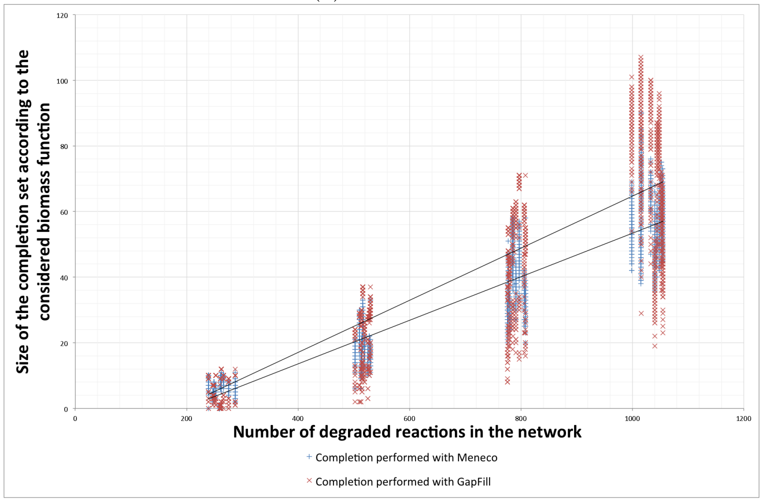

Fig 3. TCA degradation in *E. coli* degraded GEMs.

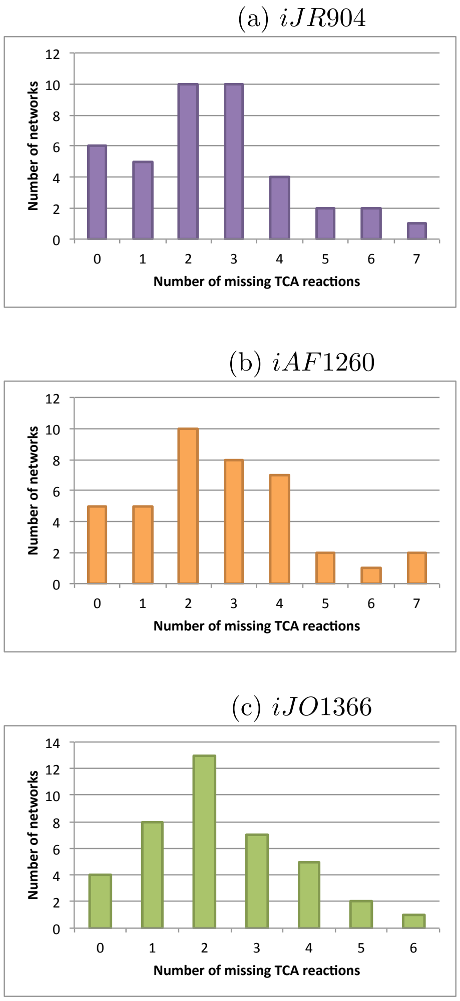

3 Restoration of biomass production by parsimonious methods

Biomass production by networks filled with Meneco and GapFill tools The capacity of the completed models to produce biomass was tested with a stoichiometry-based approach: Flux Balance Analysis (FBA). Results are shown in Fig. 4. They evidenced that Meneco was capable of restoring biomass production for 3,488 of the 10,800 degraded networks (32.3%) while GapFill restored the biomass production of 2,338 networks (21.6%).

A major difference between Meneco and GapFill can be observed for 10%-degraded networks: Meneco succeeded in restoring biomass production for 2,209 of the 2,700 degraded networks (81.8%) while GapFill restored the biomass production of 1,334 (49.4%) degraded networks. Based on the aforementioned comparative analysis of Tier 1-3 bacterial networks with different levels of manual curation, we noticed that a 10% degradation rate can be considered realistic for genomes and transcriptomes produced by NGS technologies. Thus, our analysis suggests that Meneco is a relevant tool for the preliminary completion of a new draft metabolic network when limited phenotypic information is available.

The results also suggest that the quality of the network has a strong impact on the performance of Meneco and GapFill: For the $iJR904$ network, both methods restored biomass production in 85 (2.3%) cases and they both failed in 2,602 (72.2%) cases. Meneco succeeded while GapFill failed in 890 (24.7%) cases whereas Meneco was outperformed by GapFill in 23 (0.6%) cases among the 3,600 cases (Fig. 4 (a)). For the $iAF1260$ and the $iJO1366$ networks, the capabilities of Meneco and GapFill are much more comparable (Fig. 4 (b) and (c)). This could be explained by the fact that $iAF1260$ and $iJO1366$ networks are of higher quality and robustness than $iJR904$, in turn suggesting that the capability of GapFill to restore biomass production depends on the network stoichiometry and topology whereas Meneco is more tolerant to inconsistencies.

Fig 4. Impact of Meneco and GapFill on the restoration of biomass production. Percentages and numbers of degraded networks capable of producing biomass after gap-filling with Meneco and GapFill for three different initial reference networks ($iJR904$, $iAF1260$, $iJO1366$). Among the complete benchmark of networks degraded with a rate of 10%, Meneco restored the biomass production of 81.8% of networks, while GapFill restored the biomass production of 49.4% of networks.

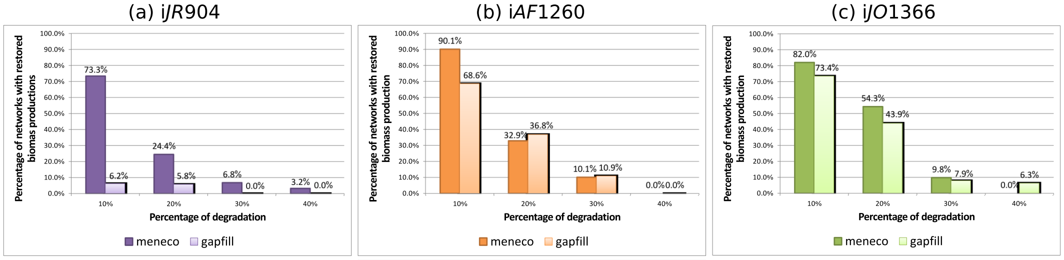

(a) $iJR904$

Number of functional networks after gap-filling	gapfill failed	gapfill succeeded	Total
meneco failed	2602	23	**2625**
10% degradation	240	0	**240**
20% degradation	652	23	**675**
30% degradation	839	0	**839**
40% degradation	871	0	**871**
meneco succeeded	890	85	**975**
10% degradation	604	56	**660**
20% degradation	196	29	**225**
30% degradation	61	0	**61**
40% degradation	29	0	**29**
**Total**	**3492**	**108**	**3600**

(b) $iAF1260$

Number of functional networks after gap-filling	gapfill failed	gapfill succeeded	Total
meneco failed	2304	98	**2402**
10% degradation	89	0	**89**
20% degradation	527	77	**604**
30% degradation	788	21	**809**
40% degradation	900	0	**900**
meneco succeeded	250	948	**1198**
10% degradation	194	617	**811**
20% degradation	42	254	**296**
30% degradation	14	77	**91**
40% degradation	0	0	**0**
**Total**	**2554**	**1046**	**3600**

(c) $iJO1366$

Number of functional networks after gap-filling	gapfill failed	gapfill succeeded	Total
meneco failed	2119	166	**2285**
10% degradation	104	58	**162**
20% degradation	411	0	**411**
30% degradation	761	51	**812**
40% degradation	843	57	**900**
meneco succeeded	297	1018	**1315**
10% degradation	135	603	**738**
20% degradation	94	395	**489**
30% degradation	68	20	**88**
40% degradation	0	0	**0**
**Total**	**2416**	**1184**	**3600**

Predicted FVA values for Meneco and GapFill tools When observing the predicted FBA values, it is interesting to note that Meneco and GapFill gap-fillings have similar results when they both restore functionality for a network. More precisely, evaluating reactions statuses with FVA after gap-filling towards biomass production was a way to compare the newly functional networks to the initial one, before degradation. To make the understanding easier, some status changes are illustrated on a simple network (Fig. 5): essential to essential, blocked to blocked, alternative to essential, alternative to blocked. Note that these status changes can only be assessed for functional networks *i.e.* with a restored biomass production.

Predicted growth values for networks gap-filled with Meneco and GapFill For each of the 10,800 degraded networks, the biomass production restoration was assessed after Meneco or GapFill gap-filling. If the predicted value for biomass production was $> 10^{-5}$ it was classified as positive. The predicted growth values were plotted if there were positive (Fig. 6). Initial biomass productions values ranged from 8188 ($iAF1260$) to 10756 mmol/gDW/hr ($iJO1366$) (7). The minimal value of biomass production was 22.6 (0.2% of the initial growth value) and the maximal one was 318061.4 (3120% of the initial biomass), both for an $iJR904$ reconstructed network.

Note that when both tools restored biomass production, the corresponding growth values were almost identical.

Table 7. Predicted biomass production values for initial networks and degraded networks gap-filled with either Meneco or GapFill.

Network	FBA value before degradation (mmol/gDW/hr)	mean FBA value after Meneco gap-filling (mmol/gDW/hr)	mean FBA value after GapFill gap-filling (mmol/gDW/hr)
$iJR904$	10,196.1	12,131.8	13,994.3
$iAF1260$	8,188.7	9,242.3	8,553.1
$iJO1366$	10,756.1	9,982.4	9,537.3

4 Overlap of Meneco and GapFill unions of solutions

We performed additional analysis experiments to asses whether Meneco and GapFill are complementary or if their results overlap each other. These experiments were done at two different scales. First we studied whether the networks for which both tools restored biomass production were similar, and compared them to the obtained networks when gap-filled with the merge solutions of both tools. Secondly we studied the reactions in the solutions of both tools to assess whether solutions were similar.

Biomass production restoration when gap-filling with the solutions of Meneco + GapFill We performed FBA experiments on networks gap-filled with the union of solutions from both Meneco and GapFill. We compared the number of networks with restored biomass production with Meneco and GapFill solutions independently and with the union of both. Fig.7 depicts the results. For $iJO1366$ and $iAF1266$ networks, there is only a small added-value to the union of both tools solutions, 119 and 85 newly functional networks respectively. However, for $iJR904$ the added-value is higher as 286 new networks become functional.

Moreover, if we study the general behaviors of the two tools, we can distinguish two cases: Meneco and GapFill both fail/succeed in restoring biomass production, or one fails and the other succeeds, the latter corresponding to a definition of complementarity. The opposite behavior of the two tools is seen in 913 (25.4%) gap-filling cases for $iJR904$, 348 for (9.7%) $iAF1260$ and 463 (12.9%) for $iJO1366$ (Fig.7). Thus with regards to

Fig 5. Possible FVA status changes after completion towards initial network The figure depicts a simple network, with compounds as circles and reactions as arrows. Essential reactions are green, alternative ones orange and blocked ones are red. No FVA can be performed on the degraded network (grey arrows) as biomass production is no longer possible. After gap-filling, R_1 stays essential and R_5 stays blocked. However, the former alternative R_6 ans R_7 became essential. The former alternative R_2 became blocked.

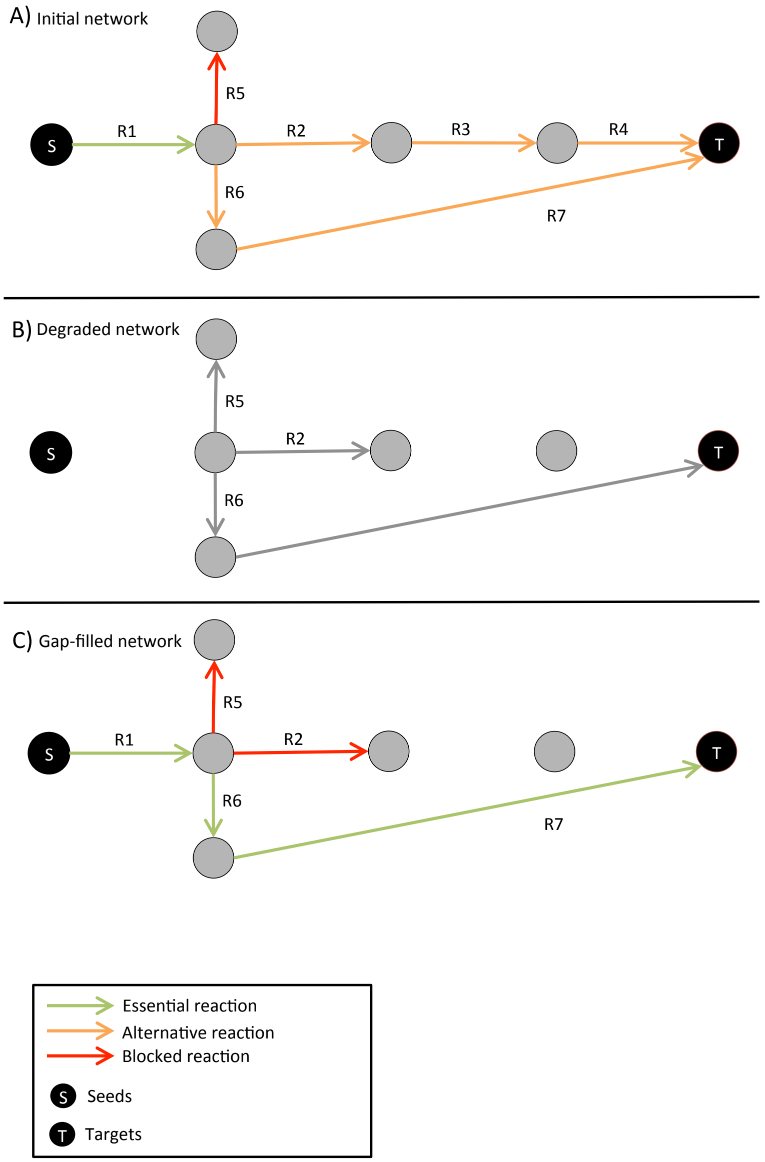

Fig 6. Predicted biomass production values for networks gap-filled with either Meneco or GapFill. The line depicts the natural logarithm of the biomass production before network degradation

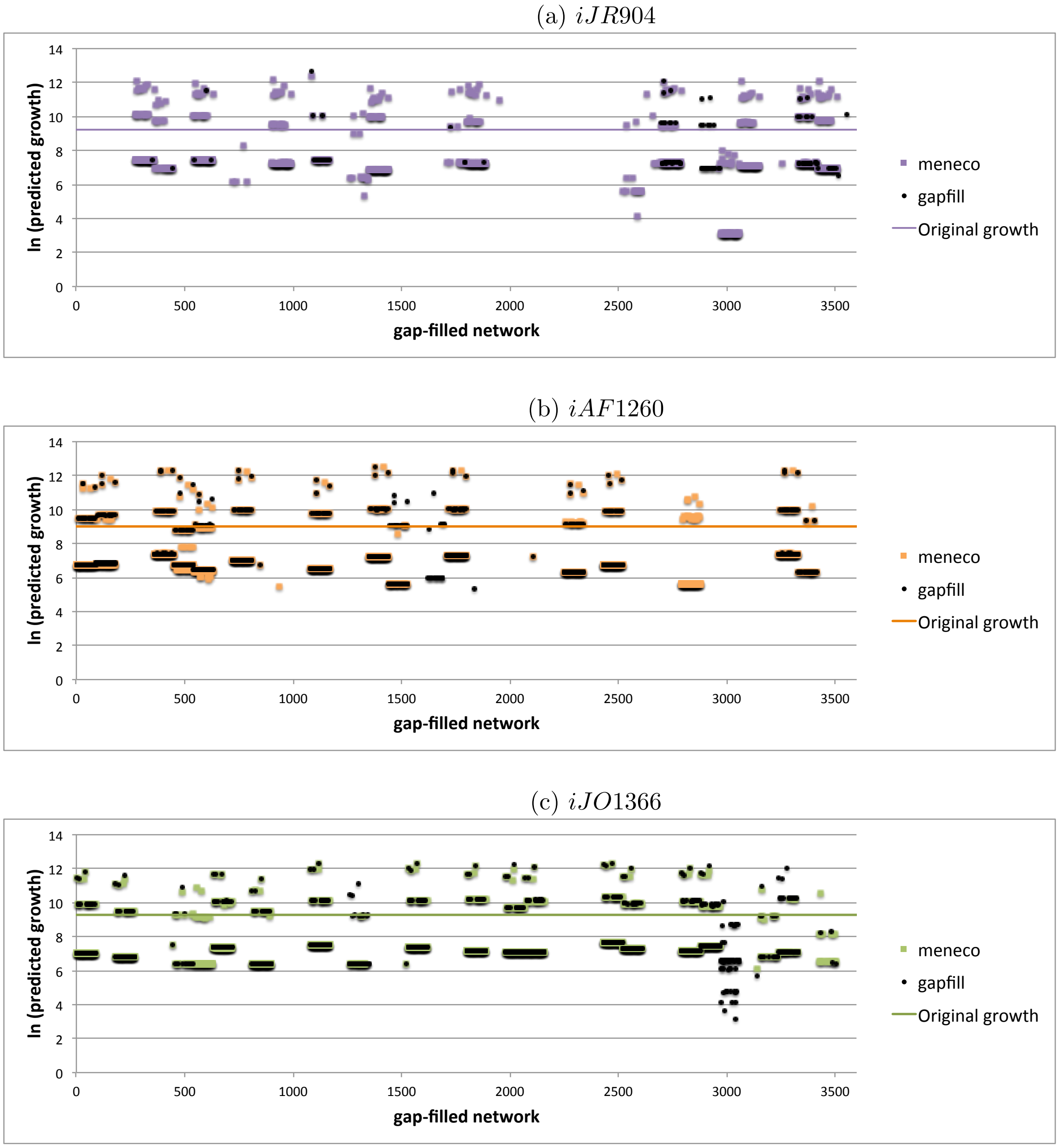

biomass production restoration, this confirms the existence of a slight complementarity between Meneco and GapFill especially regarding $iJR904$.

Study of the reactions from the solutions of Meneco + GapFill The union of Meneco and GapFill solutions (*i.e.* the union of Meneco solutions + the union of GapFill solutions) share 45.3% of their reactions (from 37% for $iJR904$ completions to 54% for $iAF1266$ networks) (Fig. 8). We then studied in detail the role of these reactions with respect to the restoration of the biomass production. We classified the reactions according to the tool it was found by: Meneco, GapFill or both. We distinguished 4 cases of FBA results: biomass production restored by Meneco, GapFill, both or none. For $iJO1366$ networks, the reaction distribution among the 3 classes was alike regardless the biomass production status: 47% of the reactions were found by Meneco and GapFill, 34% by GapFill only and 19% by Meneco. The trend was similar for $iJR904$ degraded networks, but in the cases where only GapFill restored biomass production, the percentage of reactions found only by this tool rose at the expense of those found only by Meneco. Results were more contrasted for iAF1260 networks. The percentage of reactions shared by both tools rose to 70% when they both restored biomass production, with only 3% of reactions that were found by Meneco and not by GapFill. This indicates that the union of GapFill solutions included almost all reactions of the union of Meneco solutions. When only Meneco restored biomass production, the percentage of reactions found only by this tool rose to 41%, and 43% of reactions found by both tools. This means that 85% of the reactions belonging to the union of both tools solutions were found by at least Meneco.

5 Impact of the reference database

In addition, we focused on the $iJR904$ network to evaluate the impact of the reference database. To this aim, the identifiers of $iJR904$ (BiGG database) were manually mapped to identifiers of the MetaCyc database. This allowed us to use three different repositories as reference databases for Meneco: the set of reactions from $iJR904$ itself (as in the previous analyses), the MetaCyc database (version 18.5) [2] and the union of both repositories. The results shown in Fig. 9 suggest that the MetaCyc database contains enough information about metabolic transformations to restore the biomass production of the 3,600 $iJR904$ degraded networks. Nonetheless, the $iJR904$ network and the MetaCyc database appear to contain complementary production pathways since their union enables a gap-filling that restores biomass producibility for 20.8% of the 40%-degraded networks, whereas considering either $iJR904$ or MetaCyc as a reference database restored the functionality of very few networks (3.2% and 6.7%).

These results show that the performance and the accuracy of gap-filling methods are very dependent on the content and quality of the reference database. This is a major drawback for the study of real-world problems where missing reactions may not be among well-known metabolic pathways. Based on this consideration, it should be acknowledged that the size of gap-filling completions cannot be considered as a measure of accuracy of the method.

6 Recovery of essential reactions of a metabolic network

Assessing the individual function of reactions in a network In order to investigate the reasons for failures in biomass production after gap-filling with Meneco and GapFill, reactions removed from *E. coli* networks $iJR904$, $iAF1260$ and $iJO1366$

Fig 7. Overlap of Meneco and GapFill solutions with regards to biomass production restoration Venn diagrams were generated using `http://bioinformatics.psb.ugent.be/webtools/Venn/` website.

(a) $iJR904$

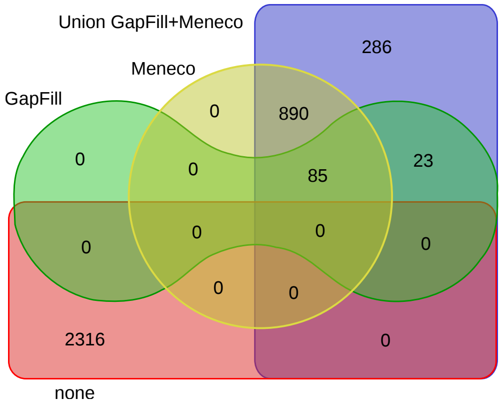

(b) $iAF1260$

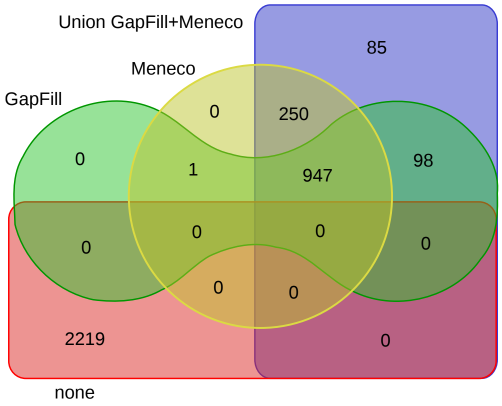

(c) $iJO1366$

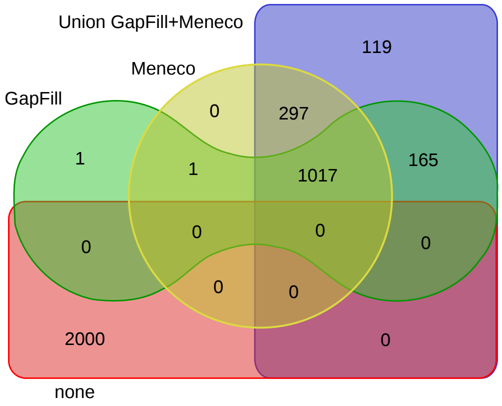

Fig 8. Reactions from Meneco and GapFill solutions X-axis depicts the FBA result towards biomass production. Y-axis depicts the percentage of reactions found either by GapFill only, by Meneco only or by both tools simultaneously.

(a) $iJR904$

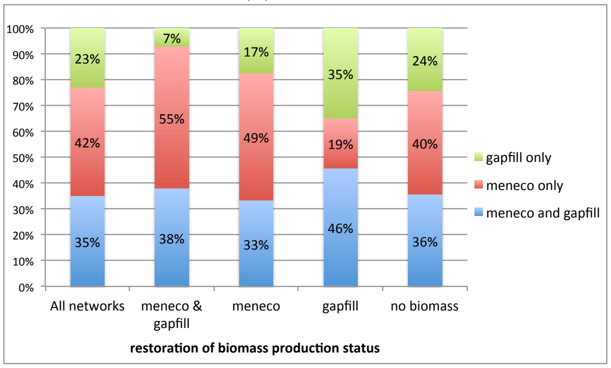

(b) $iAF1260$

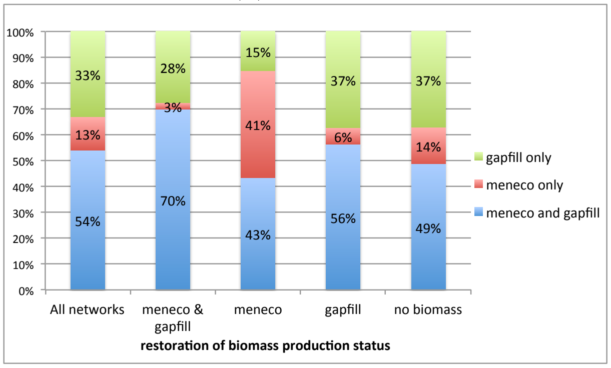

(c) $iJO1366$

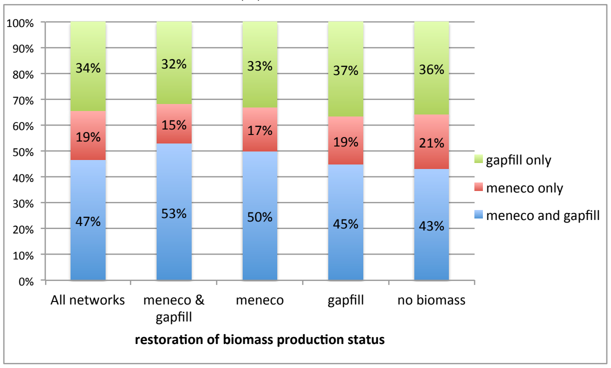

Fig 9. Impact of the reference database on the gap-filling procedure with Meneco The Meneco tool was applied to 3,600 pairs consisting of a degraded $iJR904$ *E. coli* metabolic networks (40 different networks with levels of degradation indicated by the abscissa) and a random biomass reaction (90 different reactions). The tool was used with different reference databases. The percentages of functional networks after completion is indicated on the ordinate axis.

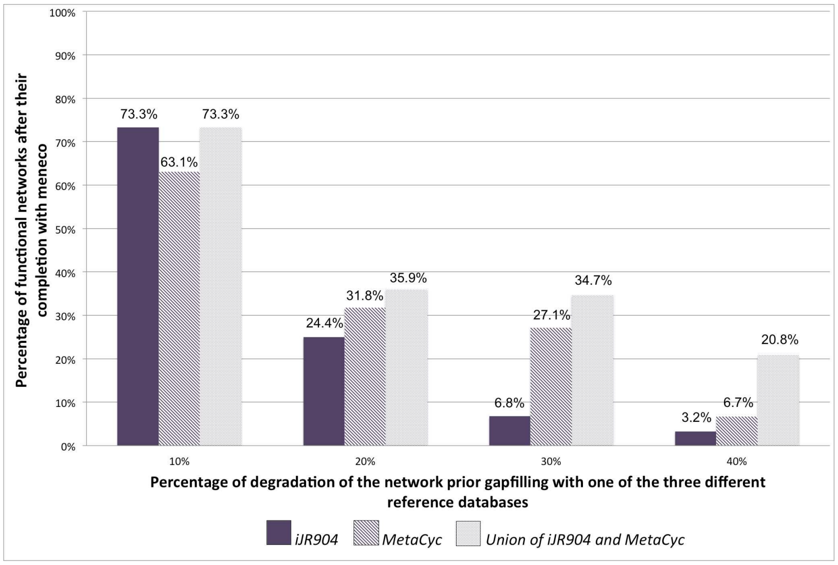

were classified according to their functionality with respect to biomass production. A reaction r was defined to be *essential* when a non-zero biomass production implied a non-zero flux through reaction r. Otherwise, if a pathway can produce the biomass without involving reaction r, then the latter is considered *alternative*. Finally, if the flux through reaction r is always zero, this reaction was classified as *blocked*. Please note that the classification of reactions into essential, alternative and blocked is highly dependent on the corresponding biomass reaction. Importantly, the degradation of *E. coli* networks carried out in our benchmarks was such that essential, alternative and blocked reactions, with respect to each of the 90 different biomass functions, were uniformly removed from the initial network.

Identification of essential reactions by Meneco and GapFill. For each of the three reference networks, the reactions of the 40 initial degraded networks were classified according to their functionality with respect to the production of their associated 90 random biomass functions. According to this classification, we tested how many essential, blocked and alternative reactions were recovered in the networks filled by the Meneco and the GapFill procedures. The results in Fig. 10 show that Meneco was able to recover most essential reactions (average recovery rate of 88.2% among the 10,800 experiments) and few blocked reactions were included in the networks filled by this tool. We also noted that Meneco often failed to recover alternative routes. Recovery rates of blocked and alternative reactions were similar for GapFill although it was less efficient than Meneco with respect to the recovery of essential reactions. Fig. 10 shows that the rate of success of both GapFill and Meneco is strongly related to the number of essential reactions removed from the network. Both methods performed equally well when 0 to 10 of these reactions were missing. Then the performance of GapFill

decreased while that of Meneco remained constant. This suggests that parsimonious topological gap-filling by Meneco is sufficient to recover essential reactions of a network even when it is highly degraded.

Fig 10. Ratios of essential reactions recovered after gap-filling by Meneco and GapFill. For each completion of a network with respect to the components of a biomass function, we estimated the capability of Meneco and GapFill to recover reactions classified in functional classes (essential, blocked, alternative). The total ratio of recovered reactions in each class was then computed. In the top panels, the average ratios of essential reactions recovered by Meneco (dark lines) and GapFill (light lines) among the 40 degraded networks are depicted for each of the 90 biomass functions represented on the X-axis. The bottom figures depict the minimal (fine dots), average (dashes), and maximal (dots) values of essential reactions missing in each considered degraded network.

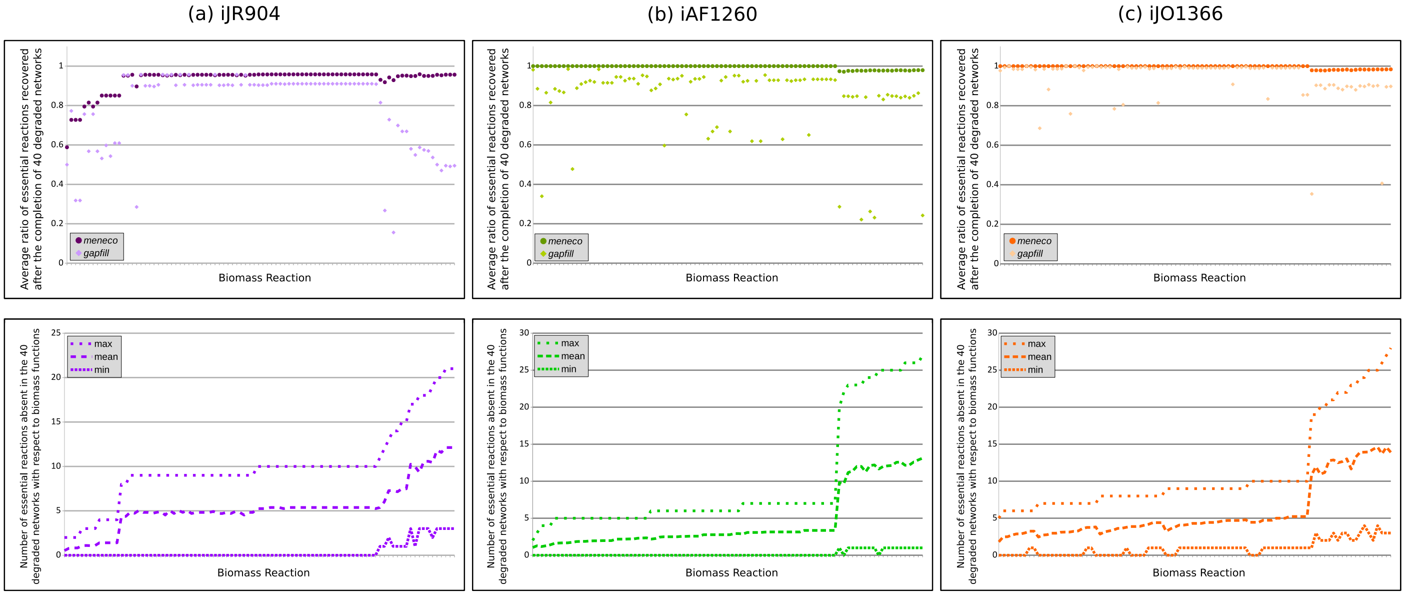

Failure to restore biomass production in FBA is mainly explained by the loss of alternative pathways To gain better insight into the importance of essential and alternative reactions in the gap-filling procedure, we classified each gap-filling experiment according to four categories: (i) the network is functional after gap-filling (ii) the network has recovered all essential reactions after gap-filling but it is not functional (iii) the gap-filling procedure missed one essential reaction and (iv) the gap-filling procedure missed more than one essential reaction. Results are depicted in Fig. 11. They confirm that in 4,050 completions (37,5%), both Meneco and GapFill recovered all essential reactions of the reference networks but nevertheless failed to restore network functionality. The loss of biomass producibility can be explained by the fact that the gap-filling procedures failed in identifying sets of alternative reactions. This was confirmed by analyzing the status of reactions in the 3,488 networks reconstructed with Meneco and capable of producing biomass: among the reactions that were essential in the reconstructed network, average 40% were classified as alternative in the reference network (see Supporting information S3). Similarly, 47% of the blocked reactions in a reconstructed network were classified as alternative in the reference

network. This suggests that the tool could be improved by relaxing the optimization scores based on parsimony assumptions.

Another interesting feature is that Meneco often missed a single essential reaction, and at most 3 in only 90 cases (0.8%). In contrast, GapFill missed more than one reaction (and up to 21) in 1,505 cases (13.9%), especially for highly degraded networks (Fig. 11).

Fig 11. Comparison of essential reactions missed during the completion of 10,800 degraded networks by Meneco and GapFill. For the 10,800 degraded $iJR904$ (purple), $iAF1260$ (orange) and $iJO1366$ (green) networks, the gap-filling results were classified according to their status: (i) restore biomass production, (ii) recover all essential reactions (dots), (iii) miss exactly one essential reaction (horizontal stripes) and (iv) miss more than one essential reaction (vertical stripes). Dark or light patterns depict the results associated with Meneco or GapFill, respectively. For each reference network, the figure depicts the size of the 12 intersections between the sets of networks sharing the same status according to both tools.

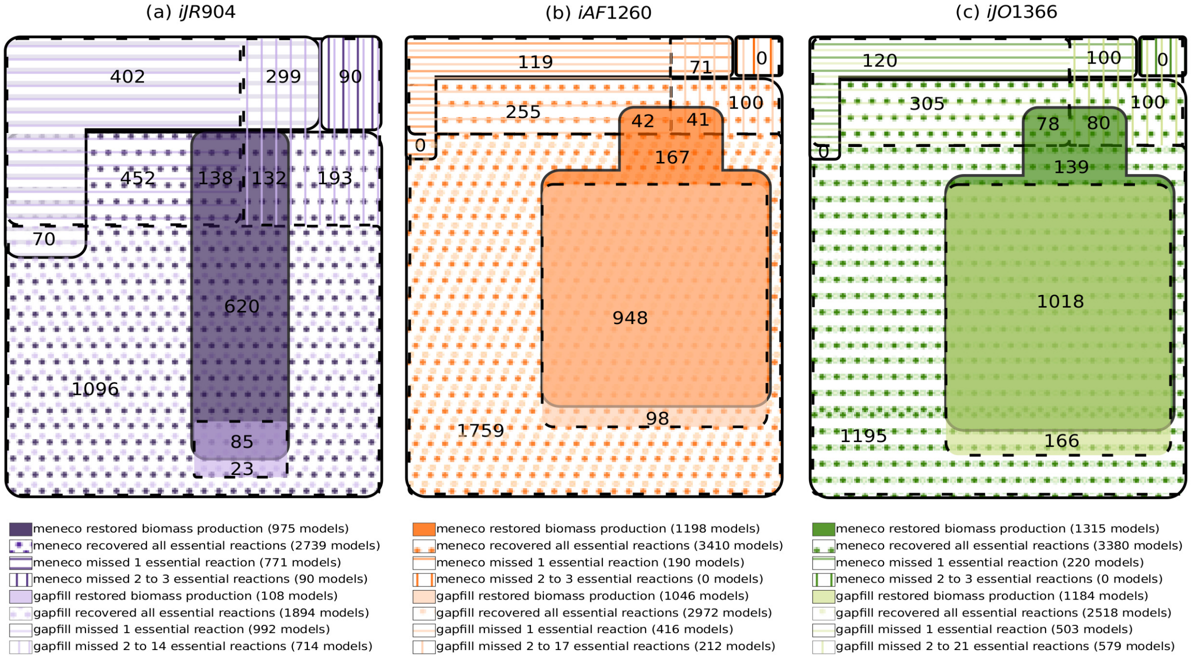

References

1. Latendresse M. Efficiently gap-filling reaction networks. BMC Bioinformatics. 2014;15(1):225. Available from: `http://www.biomedcentral.com/1471-2105/15/225`.

2. Caspi R, Altman T, Billington R, Dreher K, Foerster H, Fulcher CA, et al. The MetaCyc database of metabolic pathways and enzymes and the BioCyc collection

of Pathway/Genome Databases. Nucleic Acids Research. 2014;42(D1):D459–D471. Available from:
http://nar.oxfordjournals.org/content/42/D1/D459.abstract.
