# Supplementary material for: Meneco, a Topology-Based Gap-Filling Tool Applicable to Degraded Genome-Wide Metabolic Networks
Source: PLoS Comput Biol. 2017 Jan 27;13(1):e1005276. doi: 10.1371/journal.pcbi.1005276 (PMC5302834; doi:10.1371/journal.pcbi.1005276)
Supplement: S2 Files — The two draft networks used to study potential cross-feeding relations between E. siliculosus and Ca. P. ectocarpi are provided together with the list of seeds and targets used to run the Meneco tool. The exhaustive analysis of essential reactions which allow the production of 83 target metabolites thanks to the combination of E. siliculosus and Ca. P. ectocarpi networks is provided in a separate file. Finally, detailled examples of false positive predicted interactions and their explanation are provided in a separate pdf file. (ZIP) [file pcbi.1005276.s004.zip › Supplementary_Ectocarpus/suppEctocarpus.pdf]

Supplementary material

Examples of false positive interactions

Completion of the algal-bacterial metabolic network lead to some false positive interactions, i.e. candidate algal-bacterial interactions either due to missing gene annotations or false/overly precise annotations. We give here two of such examples, one missing annotation in the algal genome leading to wrongly assume that the bacteria was needed to provide riboflavin, and one wrong assignment of EC numbers to an algal gene leading to predict a reaction involved in peptidoglycans synthesis.

The first case (37 targets; 45% of the cases), *i.e.* the impact of missing annotations, is best illustrated by the case of riboflavin. In the algal network, riboflavin as well as several of its derivatives (FMN, $FMNH_2$, FAD, $FADH_2$), could not be produced because a RIBOPHOSPHAT-RXN reaction was not found. This phosphatase reaction, however, is only poorly characterized; relevant genes have so far only been identified in *E. coli.* It is thus probably carried out by one of the many phosphatases present in the *E. siliculosus* genome and this type of reaction should typically be added by a gap-filling method, as done by Meneco in the context of the analysis of the algal network. In *Ca.* P. ectocarpi, homologs of the *E. coli* phosphatase were found, and the corresponding reaction was predicted. Without manual validation this analysis would have therefore indicated that bacteria may provide riboflavin to the alga. Riboflavin, is a substrate for the synthesis of FAD, a co-factor in a number of other algal reactions, falsely giving the impression that a large number of algal targets depends on the metabolic input of the bacterium. One of the strong points of Meneco with this respect is that it directly identifies essential reactions necessary to produce targets in the network. During the manual curation of the Meneco results, it was therefore possible to group together all targets that require riboflavin and the RIBOPHOSPHAT-RXN, and to treat them all at the same time.

An example for the second case (21 targets; 25% of the cases), *i.e.* the effect of false or overly precise annotations, is UDP-N-acetyl-α-D-muramoyl-L-alanyl-γ-D-glutamyl-meso-2,6-diaminopimeloyl-D-alanyl-D-alanine. This compound is an intermediate in the synthesis of peptidoglycans (bacterial cell wall polysaccharides) and was added to the list of targets because the PHOSNACMURPENTATRANS-RXN (E.C. 2.7.8.13) reaction was predicted in the genome and the corresponding gene expressed in the algal network. The corresponding gene (Esi0111_0044), however, is most likely to correspond to EC 2.7.8.15, and has been annotated as such. The reason this gene was also associated with EC 2.7.8.13 in the algal network appears to be a mistake during the manual assignment of the GO term. Thus, despite the results obtained with Meneco, so far there is no indication that brown algal cell walls contain peptidoglycans, or that brown algae rely on bacteria to produce them.
