# Supplementary material for: Meneco, a Topology-Based Gap-Filling Tool Applicable to Degraded Genome-Wide Metabolic Networks
Source: PLoS Comput Biol. 2017 Jan 27;13(1):e1005276. doi: 10.1371/journal.pcbi.1005276 (PMC5302834; doi:10.1371/journal.pcbi.1005276)
Supplement: S2 Files — The two draft networks used to study potential cross-feeding relations between E. siliculosus and Ca. P. ectocarpi are provided together with the list of seeds and targets used to run the Meneco tool. The exhaustive analysis of essential reactions which allow the production of 83 target metabolites thanks to the combination of E. siliculosus and Ca. P. ectocarpi networks is provided in a separate file. Finally, detailled examples of false positive predicted interactions and their explanation are provided in a separate pdf file. (ZIP) [file pcbi.1005276.s004.zip › Supplementary_Ectocarpus/whole_table.pdf]

Local compound ID	Metacyc ID	Poducible by Ectogem ?	Producible by holobiont network ?	No. Essential reactions	Local ID of essential reactions	Metacyc Ids of essential reactions	Relevance of prediction / result of manual curation	Classification of errors	Conclusion
META23523	AGMATHINE	no	yes	1	META55371	ARGDECARBOX-RXN	Possibly: *E. siliculosus* encodes two genes involved in agmatine degradation (synthesis of polyamines), but cannot produce agmatine.	_	possible interaction
META18647	4-FUMARYL-ACETOACETATE	no	yes	1	META55928	MALEYLACETOACETATE-ISOMERASE-RXN	Possibly: The *E. siliculosus* genome encodes the complete tyrosine degradation pathway except for one step, EC 5.2.1.2. The *Ca.* P. ectocarpi genome comprises this reaction.	_	possible interaction
META21813	HISTIDINAL	no	yes	1	META50465	HISTIDPHOS-RXN	Possibly: EC 3.1.3.15 is also missing in other *Ectocarpus* strains and in *Saccharina*, but a corresponding enzyme was found in diatoms. Brown algae may rely on external histidine or histidinol, posssibly from bacteria.	_	possible interaction
META21814	HISTIDINOL	no	yes	1					
META22289	UROCANATE	no	yes	1					
META23618	HIS	no	yes	1					
META29176	AMINO-OH-HYDROXYMETHYL-DIHYDROPTERIDINE	no	yes	0*	_	_	Possible: These compounds are intermediates in tetrafolate biosynthesis. The last step in this pathway (EC 1.5.1.3) is encoded in the algal genome, but EC 6.3.2.12 is missing and may be provided by the bacterium.	_	possible interaction
META29204	DIHYDROPTERIN-CH2OH-PP	no	yes	0*					
META29211	7-8-DIHYDROPTEROATE	no	yes	0*					
META20387	CPD-597 = N-carbamoylputrescine	no	yes	0	_	_	Possibly: This compound can be produced from agmatine via the activity of an agmatine deaminase (EC 3.5.3.12, Esi0055_0036) and further converted to putrescine (EC3.5.1.53, Esi0030_0077). However, as mentioned above, an arginine decarboxylase necessary to synthesize agmatine from arginine is missing in the algal genome. The bacterium possesses a corresponding enzyme (Phect1139).	_	possible interaction
META23545	SPERMIDINE	no	yes	0	_	_	Possibly: The *E. siliculosus* genome encodes several good candidate genes for spermidine synthesis from putrescine (EC 2.5.1.16, Esi0000_0445), but putrescine synthesis in *E. siliculosus* probably relies on an external source of Agmatine (see above)	_	possible interaction
META20108	CPD-313 = propane-1,3-diamine	no	yes	0	_	_	Possibly: All compounds are related to beta-alanine synthesis. Beta-alanine is required for Vitamin B5 production in the alga and may be provided by the bacterium.	_	possible interaction
META20395	CPD-6082	no	yes	0					
META23942	B-ALANINE	no	yes	0					
META26478	CPD-330 = L-galactono-1,4-lactone	no	yes	0	_	_	Possibly: The *E. siliculosus* genome comprises several genes potentially involved in ascorbate synthesis via the L-galactose pathway (PWY-882). However, a few essential reactions are missing, notably EC 2.7.7.69, EC 1.1.1.316, and EC 2.7.7.13. *Ca.* Phaeomarinobacter is capable of producing ascorbate via the ascorbate biosynthesis pathway IV (PWY3DJ).	_	possible interaction
META30310	ASCORBATE	no	yes	0					
META29497	CPD-318 = monodehydroascorbate radical	no	yes	0					
META21893	L-DEHYDRO-ASCORBATE	no	yes	0					
META20061	CPD-237 = indole-3-acetamide	no	yes	0	_	_	Possibly: please refer to Figure 4 of the following publication of further information: http://journal.frontiersin.org/article/10.3389/fgene.2014.00241/abstract	_	possible interaction
META19429	CPD-12763 = 5-aminopentanal	no	yes	1	META50177	LYSDECARBOX-RXN	Unlikely: This compound was added to the list of targets because RXN-11784 was associated with Esi0076_0061, but the specificity of the enzyme is unknown. There is no evidence that brown algae produce biogenic amines.	_	insufficient information
META23533	Cadaverine		yes	1					
META22254	THIAMINE-P	no	yes	0*	_	_	Unlikely: Genes invoved in thiamine biosynthesis are present in the alga and the pathway is predicted. Only a cofactor required by the Thiamin-phosphate-phosphorylase, 2-(2-carboxy-4-methylthiazol-5-yl)ethyl phosphate, is not available in the alga. So far no eukaryotic and only one bacterial enzyme synthesizing this compound have been characterized. This makes a reliable identification of an algal gene based on sequence homology difficult.	_	insufficient information
META22617	CPD-9245 = palmitoleate	no	yes	10	META49353<br>META49615<br>META49359<br>META49976<br>META49402<br>META49454<br>META49305<br>META59828<br>META49619<br>META49613	RXN-10657<br>RXN-9655<br>RXN0-2145<br>RXN-10661<br>RXN-10660<br>RXN-9550<br>RXN0-2141<br>5.3.3.14-RXN<br>RXN0-2144<br>RXN-10656	None: Synthesis of these fatty acids occurs via the following pathways in the alga: PWY-5156 (not predicted; synthesis ofpalmitoyl-CoA), followed by PWY-5366/PWY-6282 (predicted/partially predicted; synthesis of palmitoleate), and PWY-5973 (partially predicted, synthesis of cis-vaccenate). Missing predictions are essentially due to the fact that not all reactions have been annotated with EC numbers in the algal genome.	Missing algal reaction due to poor annotation	probably no interaction
META22641	CPD-9245 = cis-vaccenate	no	yes	12	as above + :<br>META49820<br>META49300	as above + :<br>RXN-9555<br>RXN-9557			
META24216	C1 = UDP-N-acetyl-α-D-muramoyl-L-alanyl-y-D-glutamyl-meso-2,6-diaminopimeloyl-D-alanyl-D-alanine	no	yes	7	META59827<br>META56279<br>META52004<br>META60262<br>META50594<br>META55563<br>META54150	UDP-NACMURALGLDAPAALIG-RXN<br>UDP-NACMURALGLDAPLIG-RXN<br>UDP-NACMURALA-GLU-LIG-RXN<br>DALADALALIG-RXN<br>UDPNACETYLMURAMATEDEHYDROG-RXN<br>UDP-NACMUR-ALA-LIG-RXN<br>UDPNACETYLGLUCOSAMENOLPYRTRANS-RXN	None: These compounds are substrates for / intermediates in peptidoglycan biosynthesis. Peptidoglycans are components of bacterial cell walls but not expected in algae. They were added as targets because *E. siliculosus* expresses a gene annotated as correspondig to the PHOSNACMURPENTATRANS-RXN, which may consume/produce these compounds.However, this is most likely due to a human error during annotation.	False targets due to poor annnotation	probably no interaction
META24389	C5 = undecaprenyldiphospho-N-acetylmuramoyl-L-alanyl-y-D-glutamyl-meso-2,6-diaminopimeloyl-D-alanyl-D-alanine	no	yes	7					
META28526	CPD-9646 = di-trans,octa-cis-undecaprenyl phosphate	no	yes	0					
META22459	PALMITATE	no	yes	3	META49615<br>META49818<br>META49612	RXN-9655<br>RXN-9533<br>RXN-9537	None: All three reactions are carried out by the fatty acid synthase complex. This enzyme is probably present in *Ectocarpus*: Esi0520_0008 is a good candiate.	Missing algal reaction due to poor annotation	probably no interaction
META22460	STEARIC_ACID	no	yes	3					
META22853	DEHYDROSPHINGANINE	no	yes	3					
META22859	CPD-13612 = sphinganine	no	yes	3					
META22860	CPD-13613 = L-threo-sphinganine	no	yes	3					
META26830	PALMITYL-COA	no	yes	3					
META26885	CPD0-2117 = a long-chain trans-2,3-dehydroacyl-CoA	no	yes	3					
META18690	5-AMINO-LEVULINATE	no	yes	1	META53378	5-AMINOLEVULINIC-ACID-SYNTHASE-RXN	None: the 5-AMINOLEVULINIC-ACID-SYNTHASE-RXN is necessary to synthesize tetrapyrole from glycine, but there is no indication for the presence of the corresponding enzyme in the genome. There is an alternate pathway for production from glutamate (PWY-5188) which appears complete in *E. siliculosus*. This pathway was not automatically predicted becausea GLT -tRNA was not annotated in the genome , but it is the most probable way of Tetrapyrol biosynthesis in *E. siliculosus*.	Missing algal reaction due to poor annotation	probably no interaction
META18966	COPROPORPHYRINOGEN_III	no	yes	1					
META21833	HYDROXYMETHYLBILANE	no	yes	1					
META22102	PORPHOBILINOGEN	no	yes	1					
META28671	MG-PROTOPORPHYRIN	no	yes	1					
META28679	PROTOPORPHYRINOGEN	no	yes	1					
META28680	PROTOPORPHYRIN_IX	no	yes	1					
META28681	UROPORPHYRINOGEN-III	no	yes	1					
META28673	COPROPORPHYRINOGEN_I	no	yes	2	META53378<br>META55357	5-AMINOLEVULINIC-ACID-SYNTHASE-RXN<br>RXN-14396			
META28675	CPD-11444 = a porphyrin	no	yes	2					
META23249	CPD-10330 = α-D-ribofuranose = ribose-1-phosphate	no	yes	1	META54065	RXN-14904	This reaction most likely occurs spontaneously but had not been included in the *E. siliculosus*metabolic network.	Other	probably no interaction
META26743	ISOVALERYL-COA	no	yes	1	META62215	2KETO-4METHYL-PENTANOATE-DEHYDROG-RXN	None: A good candidate gene (Esi0000_0413) for the missing reaction was found in *E. siliculosus*, but was not been annotated accordingly.	Missing algal reaction due to poor annotation	probably no interaction
META28546	OCTAPRENYL-DIPHOSPHATE = precursor of the compound below	no	yes	1	META51463	RXN-8992 = octaprenyl/solanesyl diphosphate synthase (EC 2.5.1.90).	None: The bacterial enzyme EC 2.5.1.90 is necessary for the synthesis of Ubiquinone 8, but *E. siliculosus* possesses all genes necessary for Ubiquinone 9 synthesis (Esi0020_0133 and Esi0166_0045). These compounds were added as targets because of the association of the expressed gene Esi0552_0015 with reaction EC 2.5.1.39, but the specifictiy of the enzme is difficult to predict based on sequence information.	False targets due to poor annnotation	probably no interaction
META28521	3-OCTAPRENYL-4-HYDROXYBENZOATE	no	yes	1					
META18855	AMINO-RIBOSYLAMINO-1H-3H-PYR-DIONE	no	yes	1	META59243	RIBOPHOSPHAT-RXN	None: Phosphatases catalyzing this reaction are poorly characterized in eukaryotes and were therefore not detected in the algal metabolic network. However, several phosphatases of unknown specificity are present, and there is no compelling reason to assume that the alga relies on bacteria for FAD synthesis.	Missing algal reaction due to poor annotation	probably no interaction
META22336	CPD-12175 = (S)-3-hydroxy-isobutanoate	no	yes	1					
META26189	CH3-MALONATE-S-ALD	no	yes	1					
META26748	METHACRYLYL-COA	no	yes	1					
META26754	CPD-12173 = (S)-3-hydroxy-isobutanoyl-CoA	no	yes	1					
META29163	RIBOFLAVIN	no	yes	1					
META29164	META29164	no	yes	1					
META29165	FMN	no	yes	1					
META29166	CPD-316 = reduced riboflavin	no	yes	1					
META29167	FADH2	no	yes	1					
META29168	FMNH2	no	yes	1					
META29171	DIMETHYL-D-RIBITYL-LUMAZINE	no	yes	1					
META24487	CPD-15318 = α-D-ribose 5-phosphate	no	yes	0*	_	_	None: This compound may be produced by the alga via the activity of a ribokinase (2.7.1.15, Esi0018_0080) associated with the RIBOKIN-RXN. The product of this reaction is a compound class comprising both alpha- and beta-D-ribose 5-phosphate.	Other	probably no interaction
META26753	TRANS-3-METHYL-GLUTACONYL-COA	no	yes	0*	_	_	None: A coorsponding reaction to synthesize this compound is correctly predicted in the algal network, but lacks HCO3 as a cofactor. HCO3 is naturally present in seawater, but was not included in the list of seeds provided to meneco.	Other	probably no interaction
META18633	3OH-4P-OH-ALPHA-KETOBUTYRATE	no	yes	0*	_	_	None: There is an alternative pathway for Pyridoxal-5'-phosphate biosynthesis involving only a single reaction in the alga (PWY-6466), The corresponding reaction is catalyzed by a single enzyme EC 4.3.3.6, which is present in the *E. siliculosus*genome, but had not been annotated at the time of network reconstruction (Esi0185_0038).	Missing algal reaction due to poor annotation	probably no interaction
META18670	4-PHOSPHONOOXY-THREONINE	no	yes	0*					
META30330	PYRIDOXINE-5P	no	yes	0*					
META30326	PYRIDOXAL_PHOSPHATE	no	yes	0*					
META18930	CARBON-MONOXIDE	no	yes	0*	_	_	None: This is a byproduct of the HEME-OXYGENASE-DECYCLIZING-RXN, thought to be encoded by Esi0140_0061 in the algal genome. However, there is little evidence for the actual occurance of this reaction in the alga (enzyme specificity difficult to determine based on sequence homology).	False targets due to poor annnotation	probably no interaction
META20191	CPD-385 = 1,2-benzoquinone	no	yes	0*	_	_	None: Both compounds were added as targets based on the presence of the CATECHOL-OXIDASE-RXN (EC 1.10.3.1) in the algal network. The associated algal genes, however, correspond mainly to Tyrosinase kinases (EC 1.14.18.1) or unknown genes. "Tyrosinase kinase" is also considered a synonym for "Catechol oxidase" (EC 1.10.3.1) leading to a false association with this reaction by pathway tools.	False targets due to poor annnotation	probably no interaction
META23014	CATECHOL	no	yes	0*					
META22399	CPD-110 = salicylate	no	yes	0*	_	_	None: This target was added because it may be produced via the action of a Carboxylesterase present and expressed in the *E. siliculosus* genome. However there is no evidence of salicylate in the alga, and the specificity of the carboxylesterase cannot be determined pureley based on sequence homology.	False targets due to poor annnotation	probably no interaction
META22473	BUTYRIC_ACID	no	yes	0*	_	_	None: BUTYRIC_ACID was added to the list of targets based on the automatic annotation of the expressed gene Esi0062_0097 as putative triacylglyclerol lipase. However, this annotation is not justified due to low similarity with charaterized enzymes and as charateristic domains are missing. Butyryl-COA was included as target based on a reaction added by Pathway Tools Gapfilling, K-Hexaonyl-COA, based on the presence of an expressed enzyme who's specificity is difficult to determine based on sequence data (Esi0320_0011) and OH-HEXAONYL-COA based on the presence of an enzyme with a broad range of substrates (EC 1.1.1.35, Esi0063_0042).	False targets due to poor annnotation	probably no interaction
META26845	BUTYRYL-COA	no	yes	0*					
META26776	K-HEXANOYL-COA	no	yes	0*					
META26810	OH-HEXANOYL-COA	no	yes	0*					
META22719	OROTATE	no	yes	0*	_	_	None: This compound may be produced by the alga alone via the activity of EC 3.5.2.3. (Esi0000_0145), which has not been annotated with the corresponding EC number.	Missing algal reaction due to poor annotation	probably no interaction
META23105	PROPANOL	no	yes	0*	_	_	None: This compound was added to the list of targets based on the presence of an expressed alcohol deshydrogenase (EC 1.1.1.1) in the algal genome. The exact function of these enzymes, however is unknown.	False targets due to poor annnotation	probably no interaction
META23316	CPD-665 = propanal	no	yes	0*					
META23317	CPD-7000 = isobutanal	no	yes	0	_	_	None: Isobutanal was added as a target because RXN-7657 was predicted to be present and expressed in the alga. However, this prediction is made purely based on the presence of an alcohol dehydrogenase domain in two otherwise uncharacterized proteins.	False targets due to poor annnotation	probably no interaction
META21865	ISOBUTANOL	no	yes	0					
META22231	SUCC-S-ALD	no	yes	0	_	_	None: There is little evidence supporting the presence of this compound among the targets: the specificity of the expressed genes leading to its inclusion (4-HYDROXY-2-KETOPIMELATE-LYSIS-RXN, SUCCSEMIALDDEHYDROG-RXN) can currently not be relyably determined based on homology with charaterized sequences.	False targets due to poor annnotation	probably no interaction
META23539	CPD-14378 = dehydrospermidine	no	yes	0	_	_	None: There is very little support for the oxidation of spermidine to deshydrospermidine in *E. siliculosus* (the associated gene is poorly annotated). Thus there is no compelling reason to keep dehydrospermidine as a target.	False targets due to poor annnotation	probably no interaction
META23661	O-SUCCINYL-L-HOMOSERINE	no	yes	0	_	_	None: This compound was added to the list of targets because it is used in three reactions supported by expressed genes in the alga. In all three cases genes were associated with the reaction based on sequence homology, but are more closely realted to enzymes with other functions. We therefore currently have no evidence that o-succinyl-l-homoserine occurs in *Ectocarpus* and should be kept as a target.	False targets due to poor annnotation	probably no interaction
META23887	CPD0-2189 = 4-hydroxy-L-threonine	no	yes	0	_	_	None: This compound was added to the algal network based on the presence of an expressed gene (Esi0427_0005) associated with reaction RXN-14125 (4-phospho-hydroxy-L-threonine synthesis). This association was made automatically based on sequence homology. The enzyme corrsponding to Esi0427_0005, however, more likely constitutes a Threonine synthase.	False targets due to poor annnotation	probably no interaction

* No "essential" reactions have been identified to poduce these metabolites. At least two alternative reactions are availalbe to produce this compound.

Insufficient information

Probably no interaction - false positive

Possible interaction
