# Supplementary material for: Meneco, a Topology-Based Gap-Filling Tool Applicable to Degraded Genome-Wide Metabolic Networks
Source: PLoS Comput Biol. 2017 Jan 27;13(1):e1005276. doi: 10.1371/journal.pcbi.1005276 (PMC5302834; doi:10.1371/journal.pcbi.1005276)
Supplement: S3 Files — Both draft and functional reconstructed metabolic networks for E. mutabilis are provided here. The folder also provides the SBML file describing the repair database (MetaCyc 17.5 modified as described in the Material and Methods) required to run the Meneco tool. Files corresponding to targets and seeds are also provided. The file seed_minimum_medium_Emutabilis.sbml describes light and the 8 mineral nutrients of the minimal growth medium for E. mutabilis. As described in the main text, the network is not functional with only these seeds as reactions in cycles are blocked. The file “seeds_min_Emutabilis_final.sbml” provides the minimal set of seeds necessary to unblock the cycles and produce all the targets in “targets_Emutabilis_72.sbml” while the list of seeds and targets is also provided as tables in the file “seedsAndTargets_euglena.pdf”. (ZIP) [file pcbi.1005276.s005.zip › Supplementary_Euglene/tablesEuglena.pdf]

Supplementary material

Seeds and targets for the completion of *Euglena mutabilis* metabolic network using Meneco.

Table 1. Light and the 8 mineral seeds (shown in bold) of the minimum medium to grow *E. mutabilis* and the metabolites that needed to be added to the seeds in order to unblock the cycles in the network.

**MetaCyc id**	**metabolite**
**|Light|**	visible light
**WATER**	water
**PROTON**	H^+
**CARBON-DIOXIDE**	carbon dioxide
**SULFATE**	sulfate
**AMMONIUM**	ammonium
**Pi**	phosphate
**MG+2**	magnesium
**FE+2**	Fe^{2+}
NAD	enables 64 targets
|ACP|	enables PALMITATE and CPD-7836
|Red-Thioredoxin|	enables DCTP
|PLASTOQUINONE|	enables photosynthesis light reactions
|Oxidized-ferredoxins|	enables photosynthesis light reactions
|Oxidized-Plastocyanins|	enables photosynthesis light reactions
|RIBOSE-5P|	enables photosynthesis dark reactions and ATP production
CO-A	enables TCA and amino-acids production
|Reduced-flavodoxins|	enables DATP and DGTP production
|Ubiquinone|	enables production of pyrimidines, SUCROSE and GALACTOSE

Table 2. The 72 targets used for the completion of the *E. mutabilis* network. Targets are listed and arranged in the order in which their production by the network was addressed. The 54 metabolites that were shown to be accumulated or secreted by *E. mutabilis* are shown in bold characters.

MetaCyc id	metabolite	MetaCyc id	metabolite
OXYGEN-MOLECULE	oxygen	DATP	deoxy-adenosine triphosphate
FRUCTOSE-6P	fructose 6-phosphate	DGTP	deoxy-guanosine triphosphate
**2-KETOGLUTARATE**	2-oxoglutarate	DCTP	deoxy-cytosine triphosphate
**GLT**	glutamic acid	TTP	thymidine triphosphate
**L-ASPARTATE**	aspartic acid	**SUCROSE**	saccharose
**GLN**	glutamine	**BETA-D-FRUCTOSE**	fructose
**ASN**	arsparagine	**GALACTOSE**	galactose
L-ALPHA-ALANINE	alanine	**|D-Xylopyranose|**	D xylopyranose
**GLY**	glycine	**CPD0-1108**	D-ribofuranose
HIS	histidine	**D-LACTATE**	D lactic acid
**ILE**	isoleucine	**L-LACTATE**	L lactic acid
**LYS**	lysine	**NIACINE**	nicotinic acid
LEU	leucine	**L-THREONATE**	threonic acid
**PRO**	proline	**CYSTINE**	cystine
ARG	arginine	**ETHANOL-AMINE**	ethanol-amine
**SER**	serine	**UREA**	urea
**THR**	threonine	**|MANNOSE|**	D-mannose
**VAL**	valine	**ASCORBATE**	ascorbic acid
CYS	cysteine	**3-B-D-GALACTOSYL-SN-GLYCEROL**	3-β-D-galactosyl-sn-glycerol
**MET**	methionine	**4-hydroxybenzoate**	4-hydroxybenzoic acid
**TRP**	tryptophan	**BENZOATE**	benzoic acid
**TYR**	tyronsine	**GLYCOLLATE**	hydroxyacetic acid
**PHE**	phenylalanine	**5-OXOPROLINE**	oxoproline
**CPD-656**	Oxo-D-proline	**|D-arabinofuranose|**	D-arabinofuranose
**B-ALANINE**	β alanine	**|L-arabinofuranose|**	L-arabinofuranose
**GLYCEROL-3P**	glycerol 3-phosphate	**MANNITOL**	mannitol
**SUC**	succinic acid	**GLYCEROL**	glycerol
**GLC-6-P**	glucose 6-phosphate	**L-ORNITHINE**	ornithine
**GLC**	β D-glucose	**4-AMINO-BUTYRATE**	4-aminobutyric acid
**ALPHA-GLUCOSE**	α D-glucose	**TREHALOSE**	trehalose
**SPERMIDINE**	spermidine	**MYO-INOSITOL**	myo-inositol
**PUTRESCINE**	putrescine	CPD-7836	myristic acid
ATP	adenosine triphosphate	**PALMITATE**	palmitic acid
GTP	guanosine triphosphate	**PHYTOL**	phytol
CTP	cytosine triphosphate	CHLOROPHYLL-A	chlorophyll A
UTP	uridine triphosphate	CHLOROPHYLL-B	chlorophyll B
